# Antiretroviral drug exposure in pregnancy and risk of congenital anomalies: a European case/non-case malformed study

**DOI:** 10.1007/s00228-025-03814-w

**Published:** 2025-02-26

**Authors:** Laura Saint-Lary, Anna-Belle Beau, Agnès Sommet, Valériane Leroy, Maria Loane, Clara Cavero-Carbonell, Ester Garne, Jonathan Hoareau, Anna Latos Bielenska, Isabelle Monier, Vera Nelen, Amanda J. Neville, Mary O’Mahony, Isabelle Perthus, Anna Pierini, Anke Rissmann, Florence Rouget, Joanna Sichitiu, David Tucker, Helen Dolk, Christine Damase-Michel

**Affiliations:** 1 Inserm U1295, Team SPHERE, CERPOP, Université Paul Sabatier Toulouse 3, 37 Allées Jules Guesde, 31000 Toulouse, France; 2 Service de Pharmacologie Clinique, CHU de Toulouse, Université de Toulouse, Toulouse, France; 3 Institute of Nursing and Health Research, Ulster University, Newtownabbey, Northern Ireland UK; 4 Rare Diseases Research Unit, Foundation for the Promotion of Health and Biomedical Research in the Valencian Region, Valencia, Spain; 5 Department of Paediatrics and Adolescent Medicine, Lillebaelt Hospital, University Hospital of Southern Denmark, Kolding, Denmark; 6 Unit of Congenital Malformations, REMACOR-Medical School University of La Réunion St Pierre, Sainte-Clotilde, France; 7 Department of Medical Genetics, Poznan University of Medical Sciences, Poznań, Poland; 8 Centre for Biostatistics and Epidemiology, Paris Registry of Congenital Malformations, Obstetrical, Perinatal and Paediatric Epidemiology Research Team, INSERM, UMR 1153, Paris, France; 9 Department of Environment, PIH, Antwerp, Belgium; 10 IMER Registry (Emilia Romagna Registry of Birth Defects), Center for Clinical and Epidemiological Research, University of Ferrara, Azienda Ospedaliero Universitario Di Ferrara, Ferrara, Italy; 11 Department of Public Health, Health Service Executive-South, Cork, Ireland; 12 Department of Clinical Genetics, Centre de Référence Des Maladies Rares, Auvergne Registry of Congenital Anomalies (CEMC-Auvergne), University Hospital of Clermont-Ferrand, Clermont-Ferrand, France; 13 Unit of Epidemiology of Rare Diseases and Congenital Anomalies, Institute of Clinical Physiology, National Research Council, Pisa, Italy; 14 Medical Faculty, Malformation Monitoring Centre Saxony-Anhalt, Otto-Von-Guericke-University, Magdeburg, Germany; 15 UMR S 1085, Brittany Registry of Congenital Anomalies, University Hospital of Rennes, Rennes University, Insermaq , Irset, Rennes, France; 16 Ultrasound and Fetal Medicine Unit, Department of Woman-Mother-Child, University Hospital Center CHUV, 1011 Lausanne, CH Switzerland; 17 Congenital Anomaly Register and Information Service for Wales, Public Health Wales Knowledge Directorate, Singleton Hospital, Level 3, West Wing BlockSketty Lane, Swansea, UK; 18 School of Medicine, Ulster University, Belfast, UK

**Keywords:** Antiretroviral drug, Congenital anomalies, HIV, First trimester exposure, Pregnancy

## Abstract

**Purpose:**

Antiretroviral drugs are recommended during pregnancy to achieve HIV viral suppression and reduce mother-to-child transmission. Congenital anomaly signals were reported after fetal exposure to antiretroviral drugs in several studies warranting further investigation. We aimed to evaluate the risk of congenital anomalies after fetal exposure to antiretroviral drugs using the European congenital anomaly registry data.

**Methods:**

A case/non-case study was performed, using the EUROmediCAT central database. All the congenital anomalies, exposed to any antiretroviral drugs, were included from 1995 to 2019. We explored each signal identified from the literature for associations between congenital anomalies and specific antiretroviral exposures. We compared antiretroviral exposure between the signal anomalies (cases) and all other malformed registrations (controls). Reporting odds ratio (ROR) and their 95% confidence intervals were estimated and adjusted for registry and maternal age.

**Results:**

Between 1995 and 2019, 173 cases of congenital anomalies were observed after any exposure to antiretroviral drugs. The signal previously identified in the literature between congenital heart defects and exposure to zidovudine was confirmed in the main analysis (aROR 3.66 [1.63–8.23]). Other signals identified in the literature were not confirmed, although two cases of hypospadias and two cases of limb defects were reported after zidovudine and atazanavir exposure, respectively. The signal detection analysis did not reveal any new signal after applying the Bonferroni correction.

**Conclusions:**

Our study does not reveal new signals but confirms the previously identified signal between congenital heart defects and fetal exposure to zidovudine. The physio-pathological hypothesis induced by zidovudine exposure should be explored in future studies.

**Supplementary Information:**

The online version contains supplementary material available at 10.1007/s00228-025-03814-w.

## Introduction

In 2022, more than 38 million people were living with HIV infection, including 1.3 million pregnant women [1]. Maternal HIV infection itself is associated with adverse perinatal outcomes, such as preterm birth, low birth weight, or fetal deaths [2]. Antiretroviral drugs used during pregnancy have demonstrated a clear benefit to maternal health by inducing HIV viral suppression and preventing mother-to-child transmission (MTCT) of HIV. Since 2000, the World Health Organization (WHO) recommended antiretroviral drug regimens in pregnancy [3]. Thanks to antiretroviral drug strategies, MTCT has been reduced to less than 1%, especially in high-income countries [4, 5]. In 2019, 1.07 million pregnant women (85%) had received antiretroviral drugs for their own health, and for preventing MTCT worldwide [1].

Despite the beneficial effects of antiretroviral drugs on women’s health and the prevention of MTCT, the use of these medications still raises concerns regarding their potential adverse effects on the fetus. Several studies have reported an increased risk of adverse pregnancy outcomes after fetal exposure to some antiretroviral drugs [6–17]. In addition, congenital anomalies were observed after exposure to antiretroviral drugs: hypospadias and congenital heart defects after zidovudine exposure [18, 19]; neural tube defects after efavirenz [20–22] or dolutegravir exposure [23–25]; as well as limb and skin defects after atazanavir exposure [21]. A further study of zidovudine and congenital heart disease did not confirm an increased risk [26], and the association between efavirenz and neural tube defects has also not been confirmed in a further study [27]. In 2018, an increased number of neural tube defects related to preconceptional exposure to dolutegravir was reported. This increased risk was notified from an interim analysis of the Tsepamo study in Botswana [28]. In the final analyses, the prevalence of neural tube defects decreased from 0.9 to 0.3% (five cases of neural tube defects among 1683 pregnancies exposed to dolutegravir (0.30%, 95% CI [0.13–0.69]) [29]. Although the prevalence has declined, this remains significantly higher compared with other antiretroviral drugs. However, this risk was considered low compared with the population unexposed (one per 1000 births) [29], which does not challenge the benefit/risk balance. Consequently, since 2019, the WHO recommended dolutegravir-based regimens as the first-line treatment for HIV in all populations, including pregnant women [30–32].

These different signals between congenital anomalies and in utero exposure to antiretroviral drugs are rare fetal adverse events reported from the literature review. These risks are difficult to assess as pregnant women are usually excluded from clinical trials, and drug pharmacovigilance in pregnancy has been limited, issued from fragmented data sources [33]. These remain to be further investigated among pregnant women exposed to antiretroviral drugs using multi-source data collected after post-marketing studies. In this study, we aimed to evaluate the risk of congenital anomalies after exposure to antiretroviral drugs, using the EUROmediCAT European central database, gathering 25 congenital anomaly registries.

## Methods

We used the EUROmediCAT central database, a European research consortium dedicated to improve drug safety in pregnancy [34]. This database includes all live births, fetal deaths at 20 weeks of gestation or later, and terminations of pregnancy for fetal anomalies (TOPFA) with congenital anomalies at any gestational age. In July 2024, this database included 25 congenital anomaly registries in 17 European countries, covering more than 13 million births.

**Fig. 1:**
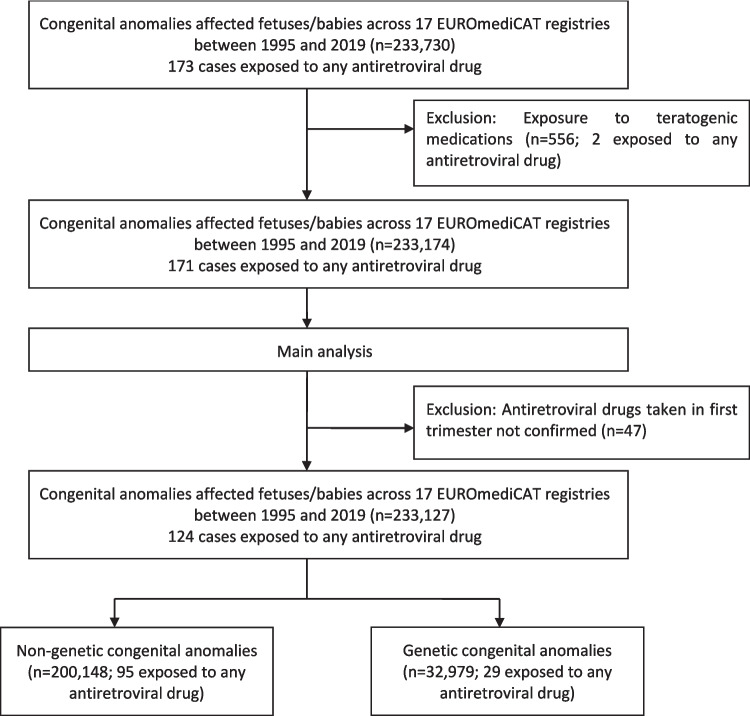
Flowchart of the study population selection in the main analyses of congenital anomalies observed in birth outcomes exposed to any antiretroviral drug from the first trimester of pregnancy in the EUROmediCAT central database between 1995 and 2019

The data provided anonymized information about the registry (center number, location), the mother (age, family history, disease diagnosed before or during pregnancy), the maternal drug exposure (drugs received during pregnancy and drugs received especially in the first trimester of pregnancy), and the baby (singleton or multiple births, gestational age at birth/termination, birth/termination year, sex, birth weight, syndromes, and congenital anomalies, age at diagnosis of congenital anomalies). All the data were collected as described in the EUROCAT Guide 1.4, the applicable guide at the time of analysis [35]. The congenital anomalies were coded according to the International Classification of Diseases ICD9 (740–759) and ICD10 (Q00-Q99) with British Pediatric Association (BPA) extension and classified into subgroups according to the EUROCAT classification. Registrations with minor congenital anomalies only according to the EUROCAT definition were excluded. Therefore, only major congenital anomalies were selected in this study. All registries participating in EUROmediCAT, which have given the approval to share and investigate data (*n* = 17), were included. The selection of the study population is summarized in Fig. 1.

### Study design

We conducted a case/non-case study [36–39] to investigate the effect of exposure to antiretroviral drugs in the first trimester of pregnancy and the occurrence of congenital anomalies, using the EUROmediCAT central database from 1995 to 2019, according to data availability at the time of study analysis as of October 2023. A signal verification analysis to investigate associations previously reported in the literature, and then a signal detection analysis to identify potential new signals between exposure to any antiretroviral drugs and specific congenital anomalies were performed.

### Study population

In this study, we included all the congenital anomalies reported in live births, fetal deaths after 20 weeks of gestation, or TOPFA, exposed to any antiretroviral drugs, from January 1, 1995, to December 31, 2019, declared in the EUROmediCAT database. Importantly, the databases do not include any data on babies/fetuses without a congenital anomaly. Consequently, we conducted a case/non-case study using malformed controls, comparing antiretroviral exposure among registrants with a specific congenital anomaly with antiretroviral exposure among all other registrants.

### Case-malformed/case-controlled definition and exposure definition

Exposed cases/non-cases with co-medications with known teratogenic effects were excluded (additional file number 1). First, for the signal verification analysis, we defined cases as all live births, fetal deaths from 20 weeks gestational age, and TOPFA with major congenital anomaly (classified by EUROCAT subgroup) which were signals identified from the literature for specific antiretroviral drug exposures, excluding those with a genetic syndrome. Non-cases were all other registrants. Second, for the signal detection analysis, all major congenital anomalies categorized by organ system were defined in turn as cases. All EUROCAT anomaly subgroups were analyzed as cases, with a changing control group consisting of all other registrants. We compared a specific congenital anomaly (“case group”) to other registrants with a diagnosis of a major congenital anomaly after the exclusion of the specific congenital anomaly being analyzed (“non-case control group”). For specific congenital anomalies, such as neural tube defects or hypospadias, we excluded other congenital anomalies in the same organ system category from the control group. Moreover, only male fetuses/babies were included in the analysis of hypospadias risk. The unexposed groups were defined for registrants without the use of any antiretroviral drugs. The control groups were classified into two categories according to the EUROCAT definition of a genetic condition (Guide 1.4): one that excluded genetic and chromosomic anomalies and one that included all non-genetic and non-chromosomal cases.

Exposure to antiretroviral drugs during the first trimester of pregnancy was defined according to the EUROCAT guide 1.4 [35] as the period from the first day of the last menstrual period to 12 completed weeks of gestation (day 0 to day 83). Antiretroviral drugs were coded according to the Anatomical Therapeutic Chemical (ATC) codes (J05A-E/F/G/R/X), reported by the World Health Organization (WHO). As the exposure period was not available for some cases included in the EUROmediCAT database, we conducted statistical analysis only in congenital anomaly cases for which maternal exposure during the first trimester was confirmed. All cases (with maternal exposure during the first trimester confirmed or not) were evaluated in a sensitivity analysis.

### Identification of congenital anomalies reported in the literature

A literature review was conducted to identify the previous congenital anomaly signals observed after exposure to antiretroviral drugs and guide our data analysis of the EUROmediCAT central database. Bibliographic searches were conducted from PubMed, Web of Science, Embase, Cochrane Library, and Reprotox. To be included, cohort studies had to have been conducted between 1995 and 2019, in high-income countries, in HIV-positive pregnant women exposed to antiretroviral drugs, in order to provide representative studies designed on data collected in the EUROmediCAT database. The inclusion criteria were similar to those applied for the population included in the EUROmediCAT central database: all congenital anomalies and all antiretroviral drugs (defined from the ATC code) were eligible.

### Data analysis

First, for all major congenital anomaly cases exposed to antiretroviral drugs, we described the distribution by registry data, maternal data (age, diseases before and during pregnancy, exposure to antiretroviral drugs), and birth data (singleton or multiple births, gestational age at birth, sex, birth weight, age at diagnosis of congenital anomalies, congenital anomalies classified by organ system). Quantitative variables were reported as mean with standard deviation, and categorical qualitative variables as numbers with percentages. Second, we conducted a signal verification study for each signal between exposure to antiretroviral drugs and a subgroup of congenital anomalies identified in the literature. Third, if less than 15 cases were exposed to a specific antiretroviral drug, an accurate description of each case, including associated genetic diagnosis, was provided (additional file number 2). Last, if there are more than 15 exposed cases of the same antiretroviral drug and more than three similar birth defects, we then conducted a signal detection analysis to detect any potential new signals.

In the signal verification study and the signal detection study, when an ATC code could not allow to identify specific antiretroviral drugs, we included these cases only in the descriptive analysis. Then, we conducted a disproportionality analysis [36–39] to estimate the reporting odds ratio (ROR) and their 95% confidence intervals (95% CI), comparing the odds of exposure in registrants with specific non-genetic congenital subgroups of anomalies (cases) to the odds of exposure among registrants with another non-genetic or genetic congenital anomalies (non-case controls), using logistic regression models. For these analyses, each antiretroviral drug was evaluated separately, the other antiretroviral drugs being excluded from the unexposed comparison group (cases and non-cases). ROR was estimated for each subgroup of congenital anomalies, defined according to the EUROCAT classification. The main analyses were performed only in congenital anomaly cases for which maternal exposure during the first trimester was confirmed. All cases (with maternal exposure during the first trimester confirmed or not) were evaluated in a sensitivity analysis. In the signal verification analysis, ROR was adjusted in logistic regression models for registry and maternal age (five categories: 14–19, 20–29, 30–40, ≥ 41 years), and we defined a significant signal of disproportionality when the lower limit of the 95% CI of the ROR was higher than one [40]. We performed a complete case analysis, namely, registrants with missing data for any of the adjustment covariates were excluded from the multivariable analyses. For the signal detection analyses, we corrected the *p*-values with the Bonferroni rule to take into account the issue of multiple testing; hence, we considered a signal to be significant if the *p*-value was below 0.0015 (as 33 comparative tests were made). Finally, when a signal was highlighted in signal detection analyses for a specific antiretroviral drug, we conducted statistical analyses for the most frequent combinations of antiretrovirals (example: tenofovir/emtricitabine). Statistical analyses were performed on Statistical Analysis Software (SAS® version 9.4).

## Results

### EUROmediCAT database description

Between 1995 and 2019, among the 233,730 fetuses/babies affected by a major congenital anomaly issued from 17 registries participating in the EUROmediCAT database, 173 registrants (0.07%) with at least one major congenital anomaly were observed after in utero exposure to any antiretroviral drug from the first trimester of gestation (Fig. 1). The majority of these exposed registrants were reported in France (50.3%), Italy (13.9%), and Spain (13.9%). HIV infection was the most frequently reported maternal morbidity before (61.8%) and during (11.6%) pregnancy, as well as hepatitis B (8.1%), obesity (6.9%), and hepatitis C (2.9%) before pregnancy, and gestational diabetes mellitus during pregnancy (2.9%). The median maternal age at delivery was 34.0 years [IQR 29.0–38.0]. There were 161 single births (93.1%) and 12 twin births (6.9%), including 50.3% male fetuses/babies. Fetal death was reported for 3.5% of exposed registrants, TOPFA for 13.9%, and live birth for 82.7%. Among live births (*n* = 143), 23.8% (34/143) were preterm births, and 32.2% (46/143) had low birth weights. All results are presented in the additional file 3.

The timing of exposure to antiretroviral drugs was confirmed to be during the first trimester of pregnancy for 124 of the exposed registrants (72.3%). Among those, the antiretroviral drugs reported in more than 20 cases were tenofovir (TDF, *n* = 65), emtricitabine (FTC, *n* = 54), lamivudine (3TC, *n* = 51), zidovudine (ZDV, *n* = 49), lopinavir (LPV, *n* = 48), abacavir (ABC, *n* = 21), and nevirapine (NVP, *n* = 20). These drugs were mostly received as triple therapy (53.8%). The results are summarized in Table 1.

**Table 1:** Description of exposure to antiretroviral drugs for the 173 cases of major congenital anomalies reported from the 17 European registries, between 1995 and 2019*

Antiretroviral drugs	173	**%**
Monotherapy	33	19.1%
Bitherapy	14	8.1%
Tritherapy	93	53.8%
Multitherapy (> 3 drugs)	8	4.6%
Unspecified	25	14.5%
Nucleoside reverse transcriptase inhibitors (NRTI)		
Tenofovir (TDF)	65	37.6%
Emtricitabine (FTC)	54	31.2%
Zidovudine (ZDV)	51	29.5%
Lamivudine (3TC)	49	28.3%
Abacavir (ABC)	21	12.1%
Stavudine (D4T)	6	3.5%
Tenofovir alafenamide (TAF)	2	1.2%
Didanosine (DDI)	2	1.2%
Zalcitabine (DDC)	1	0.6%
Non-nucleoside reverse transcriptase inhibitors (NNRTI)		
Nevirapine (NVP)	20	11.6%
Efavirenz (EFV)	3	1.7%
Rilpivirine (RPV)	2	1.2%
Etravirine (ETR)	1	0.6%
Protease inhibitors (PI)		
Lopinavir (LPV)	48	27.7%
Atazanavir (ATV)	17	9.8%
Darunavir (DRV)	15	8.7%
Ritonavir (RTV)	4	2.3%
Nelfinavir (NFV)	3	1.7%
Saquinavir (SQV)	3	1.7%
Fosamprenavir (FPV)	2	1.2%
Indinavir (IDV)	2	1.2%
Amprenavir (APV)	1	0.6%
Integrase inhibitors (INI)		
Raltegravir (RTG)	6	3.5%
Dolutegravir (DTG)	2	1.2%
Elvitegravir (EVG)	1	0.6%
Bictegravir (BIC)	1	0.6%

*All registries did not cover the same study period due to difference in data availability at the time of study analysis as of October 2023

The majority of exposed registrants had isolated congenital anomaly (64.2%). Almost half of the exposed registrants with congenital anomalies were diagnosed prenatally (46%) (additional file 3).

Congenital heart defects (42.2%), chromosomal anomalies (17.9%), limb defects (18.5%), and congenital anomalies of the urinary (11.0%) and nervous (10.4%) systems were those mainly reported after in utero exposure to any antiretroviral drug (additional file 4).

### Signals previously identified in the literature

Five previous signals were identified from the literature as summarized in Table 2.

**Table 2:** Literature signals for specific congenital anomalies after exposure to antiretroviral drugs

Congenital anomaly	Antiretroviral drug	Exposed cases (*n*)	Comparison group (*n*)	Adjusted OR/RR (95% CI)	References
Neural tube defects	Efavirenz	8/141	No-EFV 63/2842	aRR 2.56 [1.22–5.37]	[10]
		4/372	No-EFV 56/12,729	aOR 3.00 [1.10–8.50]	[41]
	Dolutegravir	5/1683	Any no-DTG 15/14,792	0.30% vs 0.1%	[29]
Congenital heart defects	Zidovudine	74/3267	No-EFV 23/2152	aOR 2.40 [1.40–4.10]	[41]
		49/3262	No-ZDV 674/9626	aOR 2.20 [1.50–3.20]	[42]
Hypospadias	Zidovudine	8/726	No-ZDV 6/1791	aOR 3.18 [1.10–9.22]	[21]
Limb defects	Atazanavir	11/222	No-ATV 46/2295	aOR 2.57 [1.30–5.08]	[21]
Skin congenital anomalies	Atazanavir	3/222	No-ATV 6/2296	aOR 6.01 [1.43–25.3]	[21]

*OR* odds ratio, *RR* relative risk.

In the studies previously cited in Table 2, the comparison groups concerned HIV-exposed uninfected children [10, 21] or deliveries [29] and live births exposed in utero to antiretroviral drugs [41, 42].

### Signal analysis

The main analysis was conducted for 124 (71.7%) cases exposed to any antiretroviral drugs, excluding two cases also exposed to the teratogenic drug (one acid valproic drug and one antimitotic drug) and 47 cases for which in utero exposure in the first trimester was not confirmed (Fig. 1).

#### Signal verification analysis (Table 3)

**Table 3:** Reporting odds ratio estimated in the main analysis for confirmation of previous signals between the occurrence of birth defects and exposure to antiretroviral drugs

Main analysis on registrants exposed during first trimester (exposure confirmed)					
Signal verification analysis	Cases (*n*, %)	Non-cases (*n*, %)		Non-cases (*n*, %)	
	Congenital heart defects (*n* = 70,425)	Other non-genetic anomalies (*n* = 129,723)	aROR [95% CI]^a^	Genetic control group (*n* = 32,979)	aROR [95% CI]^a^
ZDV	17 (0.02%)	9 (0.01%)	3.66 [1.63–8.23]	4 (0.01%)	2.45 [0.79–7.62]
	Hypospadias (*n* = 16,153)	Other non-genetic anomalies (*n* = 97,767)	aROR [95% CI]a	Genetic control group (*n* = 15,459)	aROR [95% CI]a
ZDV	2 (0.01%)	16 (0.02%)	b	43 (0.02%)	b
	Neural tube defects (*n* = 6858)	Other non-genetic anomalies (*n* = 192,296)	aROR [95% CI]a	Genetic control group (*n* = 32,979)	aROR [95% CI]a
EFV	0	1 (0.00%)	B	0	b
DTG	0	0	B	0	b
	Limb defects (*n* = 39,180)	Other non-genetic anomalies (*n* = 160,968)	aROR [95% CI]a	Genetic control group (*n* = 32,979)	aROR [95% CI]a
ATV	1 (0.00%)	9 (0.01%)	B	4 (0.01%)	b
	Skin defects (*n* = 1704)	Other non-genetic anomalies (*n* = 197,450)	aROR [95% CI]a	Genetic control group (*n* = 32,979)	aROR [95% CI]a
ATV	0	10 (0.01%)	b	4 (0.01%)	b

The non-cases were classified into two categories according to the EUROCAT definition of genetic condition (Guide 1.4): one that excluded genetic and chromosomic anomalies and one that included all non-genetic and non-chromosomal cases. The non-cases were relative to other registrants with a diagnosis of major congenital anomaly after exclusion of the specific congenital anomaly being analyzed. For specific congenital anomalies, such as neural tube defects or hypospadias, we excluded other congenital anomalies in the same organ system category from the non-cases group. We defined a significant signal of disproportionality when the lower limit of the 95%CI of the ROR was higher than one

*ATV* atazanavir, *DTG* dolutegravir, *EFV* efavirenz, *ZDV* zidovudine.

a: the RORs were adjusted on registries and maternal age.

b: ROR was calculated when more than three cases were found.

Results from the main analysis confirmed one of the five previous signals reported in the literature regarding the occurrence of congenital heart defects and fetal exposure to zidovudine, compared with the unexposed study population with a non-genetic anomaly (aROR 3.66 95% CI [1.63–8.23]). The 17 cases of congenital heart defects notified after exposure to zidovudine during the first trimester were reported between 2001 and 2015 in five countries with one to seven exposed registrants per country. All cases were live births, 11 had isolated cardiac defects, and six had associated non-cardiac anomalies. The 17 congenital heart defects notified after first trimester exposure to zidovudine were described in the additional file number 5A. In addition, two cases of hypospadias were reported after exposure to zidovudine, as well as two cases of limb defects after atazanavir exposure, not allowing to conduct a statistical analysis. Finally, across the whole dataset, no cases of neural tube defects were reported after efavirenz or dolutegravir exposure, and no cases of skin defects were reported after atazanavir exposure.

#### Signal detection analysis (Table 4)

**Table 4:** Reporting odds ratio estimated in the main analysis for detection of new signals between the occurrence of birth defects and exposure to antiretroviral drugs

Main analysis on registrants exposed during the first trimester (exposure confirmed)							
Signal detection analyses	Cases (*n*, %)	Non-cases (*n*, %)			Non-cases (*n*, %)		
	Nervous system (*n* = 20,749)	Other non-genetic anomalies (*n* = 179,399)	ROR [95% CI]	*p*-value with Bonferroni correction (*p* < 0.0015)	Genetic control group (*n* = 32,979)	ROR [95% CI]	*p*-value with Bonferroni correction (*p* < 0.0015)
TDF	6 (0.03%)	32 (0.02%)	1.89 [0.79–5.51]	0.1505	12 (0.04%)	0.91 [0.34–2.41]	0.8437
FTC	5 (0.03%)	28 (0.02%)	1.80 [0.70–4.64]	0.2263	11 (0.03%)	0.83 [0.29–2.38]	0.7251
DRV	4 (0.02%)	9 (0.00%)	4.44 [1.37–14.42]	0.0131	4 (0.01%)	1.81 [0.45–7.24]	0.4017
	Congenital heart defects (CHD) (*n* = 70,425)	Other non-genetic anomalies (*n* = 129,723)	ROR [95% CI]	*p*-value with Bonferroni correction (*p* < 0.0015)	Genetic control group (*n* = 32,979)	ROR [95% CI]	*p*-value with Bonferroni correction (*p* < 0.0015)
TDF	12 (0.02%)	26 (0.02%)	0.85 [0.43–1.69]	0.6494	12 (0.04%)	0.47 [0.21–1.04]	0.0630
FTC	11 (0.02%)	22 (0.02%)	0.92 [0.45–1.90]	0.8251	11 (0.03%)	0.47 [0.20–1.08]	0.0750
3TC	15 (0.02%)	10 (0.01%)	2.76 [1.24–6.14]	0.0129	8 (0.02%)	0.88 [0.37–2.06]	0.7600
LPV	14 (0.02%)	12 (0.01%)	2.14 [0.99–4.64]	0.0520	9 (0.03%)	0.73 [0.32–1.68]	0.4574
ABC	7 (0.01%)	5 (0.00%)	2.58 [0.82–8.12]	0.1060	5 (0.02%)	0.66 [0.21–2.06]	0.4702
ATV	5 (0.01%)	5 (0.00%)	1.84 [0.44–6.36]	0.3344	4 (0.01%)	0.59 [0.16–2.18]	0.4241
DRV	5 (0.01%)	8 (0.01%)	1.15 [0.38–3.53]	0.7409	4 (0.01%)	1.71 [0.46–6.37]	0.4241
	Severe CHD (*n* = 19,834)	Other non-genetic anomalies excluding others CHD (*n* = 129,723)	ROR [95% CI]	*p*-value with Bonferroni correction (*p* < 0.0015)	Genetic control group (*n* = 32,979)	ROR [95% CI]	*p*-value with Bonferroni correction (*p* < 0.0015)
TDF	4 (0.02%)	34 (0.02%)	1.35 [0.48–3.79]	0.5740	12 (0.04%)	0.68 [0.22–2.12]	0.5087
FTC	4 (0.02%)	29 (0.02%)	1.58 [0.56–4.50]	0.3890	11 (0.03%)	0.75 [0.24–2.34]	0.6138
	Ventricular septal defect (*n* = 38,570)	Other non-genetic anomalies excluding others CHD (*n* = 129,723)	ROR [95% CI]	*p*-value with Bonferroni correction (*p* < 0.0015)	Genetic control group (*n* = 32,979)	ROR [95% CI]	*p*-value with Bonferroni correction (*p* < 0.0015)
TDF	4 (0.01%)	34 (0.02%)	0.55 [0.20–1.54]	0.2559	12 (0.04%)	0.31 [0.10–0.96]	0.0430
FTC	5 (0.01%)	28 (0.02%)	0.84 [0.33–2.17]	0.7163	11 (0.03%)	0.42 [0.15–1.22]	0.1109
3TC	7 (0.02%)	18 (0.01%)	1.81 [0.76–4.34]	0.1821	8 (0.02%)	0.82 [0.30–2.24]	0.6902
ZDV	6 (0.02%)	20 (0.01%)	1.39 [0.56–3.47]	0.4753	4 (0.01%)	1.39 [0.39–4.92]	0.6115
LPV	7 (0.02%)	19 (0.01%)	1.72 [0.72–4.08]	0.2224	9 (0.03%)	0.73 [0.27–1.95]	0.5227
ABC	4 (0.01%)	8 (0.00%)	2.43 [0.71–7.76]	0.1649	5 (0.02%)	0.75 [0.20–2.78]	0.6609
	Digestive system (*n* = 15,633)	Other non-genetic anomalies (*n* = 184,515)	ROR [95% CI]	*p*-value with Bonferroni correction (*p* < 0.0015)	Genetic control group (*n* = 32,979)	ROR [95% CI]	*p*-value with Bonferroni correction (*p* < 0.0015)
TDF	4 (0.03%)	34 (0.02%)	1.59 [0.56–4.46]	0.3821	12 (0.04%)	0.79 [0.26–2.46]	0.6869
FTC	5 (0.04%)	28 (0.02%)	2.40 [0.93–6.22]	0.0713	11 (0.03%)	1.08 [0.38–3.11]	0.8842
3TC	3 (0.02%)	22 (0.01%)	1.85 [0.56–6.16]	0.3143	8 (0.02%)	0.89 [0.23–3.37]	0.8683
	Genital system (*n* = 19,793)	Other non-genetic anomalies (*n* = 180,355)	ROR [95% CI]	*p*-value with Bonferroni correction (*p* < 0.0015)	Genetic control group (*n* = 32,979)	ROR [95% CI]	*p*-value with Bonferroni correction (*p* < 0.0015)
TDF	4 (0.03%)	34 (0.02%)	1.12 [0.40–3.15]	0.8297	12 (0.04%)	0.58 [0.19–1.78]	0.3385
FTC	4 (0.03%)	29 (0.02%)	1.31 [0.46–3.72]	0.6153	11 (0.03%)	0.63 [0.20–1.97]	0.4249
	Urinary system (*n* = 31,378)	Other non-genetic anomalies (*n* = 168,770)	ROR [95% CI]	*p*-value with Bonferroni correction (*p* < 0.0015)	Genetic control group (*n* = 32,979)	ROR [95% CI]	*p*-value with Bonferroni correction (*p* < 0.0015)
TDF	8 (0.03%)	30 (0.02%)	1.53 [0.70–3.34]	0.2850	12 (0.04%)	0.74 [0.54–3.41]	0.5140
FTC	7 (0.02%)	26 (0.02%)	1.55 [0.67–3.56]	0.3069	11 (0.03%)	0.71 [0.28–1.83]	0.4787
	Congenital hydronephrosis (*n* = 10,695)	Other non-genetic anomalies excluding other urinary congenital anomalies (*n* = 168,770)	ROR [95% CI]	*p*-value with Bonferroni correction (*p* < 0.0015)	Genetic control group (*n* = 32,979)	ROR [95% CI]	*p*-value with Bonferroni correction (*p* < 0.0015)
TDF	3 (0.03%)	35 (0.02%)	1.58 [0.49–5.12]	0.4472	12 (0.04%)	0.80 [0.23–2.82]	0.7243
	Limb defects (*n* = 39,180)	Other non-genetic anomalies (*n* = 160,968)	ROR [95% CI]	*p*-value with Bonferroni correction (*p* < 0.0015)	Genetic control group (n = 32,979)	ROR [95% CI]	*p*-value with Bonferroni correction (*p* < 0.0015)
TDF	10 (0.03%)	28 (0.02%)	1.61 [0.78–3.31]	0.1963	12 (0.04%)	0.76 [0.33–1.75]	0.5117
FTC	10 (0.03%)	23 (0.01%)	1.96 [0.94–4.13]	0.0746	11 (0.03%)	0.82 [0.35–1.94]	0.6567
3TC	5 (0.01%)	20 (0.01%)	1.13 [0.43–3.01]	0.8062	8 (0.02%)	0.57 [0.19–1.74]	0.3244
LPV	7 (0.02%)	19 (0.01%)	1.70 [0.70–3.95]	0.2511	9 (0.03%)	0.71 [0.26–1.89]	0.4882
ABC	3 (0.01%)	9 (0.01%)	1.50 [0.41–5.55]	0.5421	5 (0.02%)	0.55 [0.13–2.29]	0.4108
NVP	4 (0.01%)	6 (0.00%)	3.02 [0.85–10.68]	0.0870	3 (0.01%)	1.21 [0.27–5.39]	0.8059
DRV	5 (0.01%)	8 (0.00%)	2.83 [0.93–8.65]	0.0677	4 (0.01%)	1.13 [0.30–4.22]	0.8534
	Polydactyly (*n* = 10,190)	Other non-genetic anomalies excluding other limb defects (*n* = 160,968)	ROR [95% CI]	*p*-value with Bonferroni correction (*p* < 0.0015)	Genetic control group (*n* = 32,979)	ROR [95%CI]	*p*-value with Bonferroni correction (*p* < 0.0015)
TDF	6 (0.06%)	32 (0.02%)	3.78 [1.58–9.01]	0.0027	12 (0.04%)	1.73 [0.65–4.61]	0.2737
FTC	5 (0.05%)	28 (0.01%)	3.57 [1.83–9.24]	0.0088	11 (0.03%)	1.57 [0.55–4.52]	0.4025
LPV	4 (0.04%)	22 (0.01%)	3.63 [1.25–10.54]	0.0177	9 (0.03%)	1.54 [0.47–4.99]	0.4753

The non-cases were classified into two categories according to the EUROCAT definition of genetic condition (Guide 1.4): one that excluded genetic and chromosomic anomalies and one that included all non-genetic and non-chromosomal cases. The non-cases were relative to other registrants with a diagnosis of major congenital anomaly after exclusion of the specific congenital anomaly being analyzed. For specific congenital anomalies, such as neural tube defects or hypospadias, we excluded other congenital anomalies in the same organ system category from the non-cases group.

*ABC* abacavir, *ATV* atazanavir, *CHD* congenital heart defects, *FTC* emtricitabine, *LPV* lopinavir, *NVP* nevirapine, *TDF* tenofovir, *ZDV* zidovudine, *3TC* lamivudine, *95% CI* 95% confidence intervals.

Exploring the EUROmediCAT database to detect associations has led to identify the additional following signal before Bonferroni correction: congenital heart defects were reported after exposure to lamivudine (ROR non-genetic group 2.76 95% CI [1.24–6.14]) (additional file number 5A). Congenital anomalies of the nervous system after darunavir exposure were found (ROR: non-genetic group 4.44 95% CI [1.37–14.42]) (additional file number 5B). Polydactyly was observed after exposure to tenofovir (ROR non-genetic group 3.68 95% CI [1.52–8.88]), emtricitabine (ROR non-genetic group 3.76 95% CI [1.43–9.87]), and lopinavir (ROR non-genetic group 3.63 95% CI [1.25–10.54]) (additional file number 5C). However, there was no longer a significant signal identified after applying the Bonferroni correction. The results were similar in the sensitivity analyses including all cases (exposure confirmed or not during the first trimester, *n* = 171; additional file number 6).

## Discussion

Associations between exposure to antiretroviral drugs during the first trimester of pregnancy and congenital anomalies were assessed from the European congenital anomalies registries, in the EUROmediCAT central database. Between 1995 and 2019, 173 cases of major congenital anomalies were observed after in utero exposure to any antiretroviral drugs in 17 registries, of which 124 exposures were confirmed as first-trimester exposures.

A previous signal between congenital heart defects and fetal exposure to zidovudine was identified in the literature [41–43] and was confirmed in our main verification signal analysis (aROR 3.66 95% CI [1.63–8.23]). This signal was reported from French studies, including 13,124 live births exposed to antiretroviral drugs during pregnancy, among which 74 and 90 congenital heart defects were identified after zidovudine exposure (74/3267 and 90/3262 cases exposed to zidovudine, respectively [41, 42] (French Perinatal Cohort and randomized trial ANRS 135 PRIMEVA). However, other studies conducted in the USA, using data from the Antiretroviral Pregnancy Registry (APR), Medicaid, or systematic reviews and meta-analyses [26, 44–47], did not align with our result. These studies included, respectively, 15,451 live birth outcomes with 36 septal defects [26] and 1,391 pregnancies with eight congenital heart defects [40] from the APR. Another study conducted from Medicaid reported an adjusted OR of 1.20 [0.58–2.51] from 1932 pregnancies [41], and two systematic reviews and meta-analyses also documented non-significant OR between exposure to zidovudine during the first trimester of pregnancy and major malformations (OR 1.30 [0.63–2.71] [42], OR 1.01 [0.30–4.95] [43]). We can suppose that the populations included in the American and French studies differed. Indeed, populations that arose from the Medicaid database presented generally specific socio-demographic conditions. In addition, the data included in the APR report are based on cohort studies, clinical trials, and malformation registries. In our study, the cases are reported from population-based congenital anomaly registries exclusively, including TOPFA, giving better reliability and validity to the results obtained. However, it is likely that the cases included in our study were also reported within the two French studies [41, 42]. The association between zidovudine exposure and cardiomyopathy may be explained by mitochondrial toxicity but needs to be further investigated, especially to also explore the association with congenital heart defects, since the origin of functional heart diseases is different from congenital heart defects [48, 49]. This could have an impact on cardiac function and, therefore, on congenital heart defects. It will be relevant to investigate the long-term effects of mitochondrial toxicity during childhood [50]. Since this risk is considered low (< 0.5%, 9 per 1000 in our study), zidovudine is always recommended in the combination of antiretroviral drugs used during pregnancy, thanks to the significant benefit related to the reduction of mother-to-child transmission of HIV.

The signal detection analysis identified additional potential signals that were not reported elsewhere. However, after Bonferroni correction, these signals were not statistically significant but deserve to keep our focus on. Congenital heart defects and ventricular septal defects were observed after exposure to lamivudine. This signal could be explained by NRTI-induced mitochondrial toxicity, like zidovudine in the same class of drug [41–43]. Polydactyly was observed after exposure to tenofovir and emtricitabine, as well as congenital anomalies of the nervous system after darunavir exposure. Some specific congenital anomalies of the nervous system, namely neural tube defects cases were identified in four pharmacovigilance databases [51] (16 among VigiBase® and 3 among Medicines Health Regulatory Authority, EudraVigilance, and FAERS Public Domain databases), without significant association, although no neural tube defect case was found in a recent French cohort study [52]. The heart and neurological signals, although not statistically significant after application of the Bonferroni correction, could impair severely fetal development and should be investigated and monitored in future pharmaco-epidemiological studies to assess the teratogenic risk of these specific antiretroviral drugs (tenofovir, emtricitabine, lamivudine, lopinavir, and darunavir).

This study presents some limitations. Some covariables were not available in the EUROmediCAT database, including maternal comorbidities (gestational diabetes, coinfection with hepatitis, etc.), dosage, or duration of drug exposure. Moreover, other potential confounders, such as viral load, CD4 counts, and maternal conditions (body mass index, substance use including drugs, tobacco or alcohol, or other maternal drug exposure were not available in the EUROmediCAT database. Indeed, the regression models were only adjusted on maternal age and registry, without taking into account the potential effect of other confounders in the association found. Drug exposure in the first trimester was not confirmed in some cases, leading to exclusion in the main analysis. However, the results of the sensitivity analyses, including all cases, were similar to those reported in the main analysis. Moreover, we selected the malformed control group in this study, which is not the most intuitive comparison group. The major benefit of this comparison group is to be derived from the same data source. Estimates might also be underestimated if malformed non-cases include congenital anomalies eventually associated with antiretroviral exposure, but this will be mitigated by the use of the genetic non-case group. However, without a control group without congenital anomaly, the ROR was relative to other major malformations and could not be generalize to the general population. Even though the analyses were conducted on a large European population, we observed small numbers of cases exposed to several antiretroviral drugs, making some analyses impossible to be carried out. As a result, we could not conclude on a potential association between efavirenz, dolutegravir, and atazanavir and specific congenital anomalies. Finally, as antiretroviral drugs are usually used in multiple therapy, it is difficult to attribute the observed adverse effect to a specific drug.

Our study has also several strengths. First, the EUROmediCAT central database provides standardized information on congenital anomalies, unlike the majority of other data sources available for the evaluation of adverse perinatal outcomes, especially congenital anomalies. The congenital anomalies are well documented, with accurate diagnoses, and include TOPFA which is generally excluded from other studies. Secondly, for most of the cases reported in the EUROmediCAT central database, drug exposure during the first trimester was confirmed. This represents a major advantage in the assessment of congenital anomalies risk related to drug exposure since the organogenesis occurred during the first trimester of pregnancy [53]. Third, the data available in the EUROmediCAT database reflect the whole population of the geographical area covered by registries, as the registrations are documented from all women and all pregnancies, including all types of congenital anomalies and medication use in pregnancy (https://www.euromedicat.eu/content/EUROmediCAT-Leaflet.pdf). Fourth, validated and standardized methods were applied. ATC codes were used to identify and confirm exposures to antiretroviral drugs, as well as ICD9-10 codes and the EUROCAT classification [35] for congenital anomalies.

## Conclusions

Our study using a European database has confirmed the previous signal identified between congenital heart defects and fetal exposure to zidovudine. To date, the benefit/risk balance remains in favor of the use of zidovudine during pregnancy, thanks to the prevention of mother-to-child transmission. With this latter exception, overall, no additional signal was detected, some further associations were found that may have been due to multiple testing. These results should be considered to better understand the risk of congenital anomalies after fetal exposure to antiretroviral drugs and improve pregnancy monitoring of HIV-positive women.

## Supplementary Information

Below is the link to the electronic supplementary material.

Supplementary file1 (DOCX 215 KB)

## Data Availability

No datasets were generated or analysed during the current study.
