# Supplementary material for: Antiretroviral drug exposure in pregnancy and risk of congenital anomalies: a European case/non-case malformed study
Source: Eur J Clin Pharmacol. 2025 Feb 26;81(5):697–709. doi: 10.1007/s00228-025-03814-w (PMC12003615; doi:10.1007/s00228-025-03814-w)
Supplement: Supplementary file 1 — Supplementary file1 (DOCX 215 KB) [file 228_2025_3814_MOESM1_ESM.docx]

Supplementary 2.0

**Additional file number 1: Description of teratogenic drugs.**

The following drugs were considered as teratogenic drugs in the literature and were excluded from the study using the ATC codes and the name:

| **ATC codes** | **Drugs** |
| --- | --- |
| N03AG01 | Valproic acid |
| N03AG02 | Valpromide |
| D05BB, D10BA, D11AH04 | Oral retinoids |
| B01AA | Oral anticoagulants |
| L04AA06 | Mycophenolate |
| L04AX02 | Thalidomide |
| L01A, L01B, L01C | Antimitotics |

**Additional file number 2: Description of congenital anomalies observed by EUROCAT subgroup and by specific antiretroviral drug (n<15), after exposure to antiretroviral drugs, in 17 European registries between 1995 and 2019*.**

|  |  |  | **Nucleoside reverse transcriptase inhibitors  (n,%)** | | | | **Non-nucleoside reverse transcriptase inhibitors  (n,%)** | | | **Protease inhibitors  (n,%)** | | | | | | **Integrase inhibitors (n,%)** | | | |
| --- | --- | --- | --- | --- | --- | --- | --- | --- | --- | --- | --- | --- | --- | --- | --- | --- | --- | --- | --- |
|  | Unexposed  to ARV | ARV | D4T | TAF | DDI | DDC | EFV | RPV | ETR | RTV | NFV | SQV | FPV | IDV | APV | RTG | DTG | EVG | BTG |
| Anomaly group | 232418 | 173 | 6 | 2 | 2 | 1 | 3 | 2 | 1 | 4 | 3 | 3 | 2 | 1 | 1 | 6 | 2 | 1 | 1 |
| Nervous system | 20841 (9.0%) | 18 (10.4) |  | 0 | 0 | 0 | 0 | 1 (50) | 0 | 0 | 0 | 0 | 0 | 0 | 0 | 1 (16.7) | 0 | 0 | 0 |
| – Neural Tube Defects | 7284 (3.1%) | 1 (0.6) | 0 | 0 | 0 | 0 | 0 | 0 | 0 | 0 | 0 | 0 | 0 | 0 | 0 | 0 | 0 | 0 | 0 |
| – – Anencephalus and similar | 2078 (0.9%) | 0 | 0 | 0 | 0 | 0 | 0 | 0 | 0 | 0 | 0 | 0 | 0 | 0 | 0 | 0 | 0 | 0 | 0 |
| – – Encephalocele | 939 (0.4%) | 0 | 0 | 0 | 0 | 0 | 0 | 0 | 0 | 0 | 0 | 0 | 0 | 0 | 0 | 0 | 0 | 0 | 0 |
| – – Spina Bifida | 4267 (1.8%) | 1 (0.6) | 0 | 0 | 0 | 0 | 0 | 0 | 0 | 0 | 0 | 0 | 0 | 0 | 0 | 0 | 0 | 0 | 0 |
| – Microcephaly | 2580 (1.1%) | 3 (1.7) | 0 | 0 | 0 | 0 | 0 | 0 | 0 | 0 | 0 | 0 | 0 | 0 | 0 | 0 | 0 | 0 | 0 |
| – Hydrocephalus | 4463 (1.9%) | 3 (1.7) | 0 | 0 | 0 | 0 | 0 | 0 | 0 | 0 | 0 | 0 | 0 | 0 | 0 | 0 | 0 | 0 | 0 |
| – Arhinencephaly/holoprosencephaly | 1000 (0.4%) | 0 | 0 | 0 | 0 | 0 | 0 | 0 | 0 | 0 | 0 | 0 | 0 | 0 | 0 | 0 | 0 | 0 | 0 |
| Eye | 4094 (1.8%) | 0 |  | 1 (50) | 0 | 0 | 0 | 0 | 0 | 0 | 0 | 0 | 0 | 0 | 0 | 0 | 0 | 0 | 0 |
| – Anophthalmos/micropthalmos | 945 (0.4%) | 0 | 0 | 0 | 0 | 0 | 0 | 0 | 0 | 0 | 0 | 0 | 0 | 0 | 0 | 0 | 0 | 0 | 0 |
| – – Anophthalmos | 209 (0.1%) | 0 | 0 | 0 | 0 | 0 | 0 | 0 | 0 | 0 | 0 | 0 | 0 | 0 | 0 | 0 | 0 | 0 | 0 |
| – Congenital cataract | 1185 (0.5%) | 0 | 0 | 0 | 0 | 0 | 0 | 0 | 0 | 0 | 0 | 0 | 0 | 0 | 0 | 0 | 0 | 0 | 0 |
| – Congenital glaucoma | 304 (0.1%) | 0 | 0 | 0 | 0 | 0 | 0 | 0 | 0 | 0 | 0 | 0 | 0 | 0 | 0 | 0 | 0 | 0 | 0 |
| Ear, face and neck | 2703 (1.2%) | 2 (1.2) | 0 | 1 (50) | 0 | 0 | 0 | 0 | 0 | 0 | 0 | 1 (33.3) | 0 | 0 | 0 | 0 | 0 | 0 | 1 (100) |
| – Anotia | 335 (0.1%) | 0 | 0 | 0 | 0 | 0 | 0 | 0 | 0 | 0 | 0 | 0 | 0 | 0 | 0 | 0 | 0 | 0 | 0 |
| Congenital heart defects | 79676 (34.3%) | 73 (42.2) | 2 (33.3) | 2 (100) | 1 (50) | 0 | 1 (33.3) | 1 (50) | 1 (100) | 0 | 0 | 2 (66.6) | 2 (100) | 1 (100) | 1 (100) | 1 (16.7) | 0 | 1 (100) | 1 (100) |
| – Severe CHD § | 19866 (8.6%) | 26 (15.0) | 0 | 0 | 0 | 0 | 0 | 0 | 0 | 0 | 0 | 0 | 0 | 0 | 0 | 0 | 0 | 0 | 0 |
| – Common arterial truncus | 649 (0.3%) | 2 (1.2) | 0 | 0 | 0 | 0 | 0 | 0 | 0 | 0 | 0 | 0 | 0 | 0 | 0 | 0 | 0 | 0 | 0 |
| – Double outlet right ventricle § | 928 (0.4%) | 1 (0.6) | 0 | 0 | 0 | 0 | 0 | 0 | 0 | 0 | 0 | 0 | 0 | 0 | 0 | 0 | 0 | 0 | 0 |
| – Transposition of great vessels | 2881 (1.2%) | 2 (1.2) | 0 | 1 (50) | 0 | 0 | 0 | 0 | 0 | 0 | 0 | 1 (33.3) | 0 | 0 | 0 | 0 | 0 | 0 | 1 (100) |
| – Single ventricle | 627 (0.3%) | 0 | 0 | 0 | 0 | 0 | 0 | 0 | 0 | 0 | 0 | 0 | 0 | 0 | 0 | 0 | 0 | 0 | 0 |
| – Ventricular septal defect (VSD) | 38634 (16.6%) | 29 (16.8) | 1 (16.7) | 2 (100) | 1 (50) | 0 | 1 (33.3) | 0 | 0 | 0 | 0 | 1 (33.3) | 0 | 1 (100) | 1 (100) | 0 | 1 (50) | 0 | 1 (100) |
| – Atrial septal defect (ASD) | 19076 (8.2%) | 12 (6.9) | 1 (16.7) | 1 (50) | 0 | 0 | 1 (33.3) | 0 | 0 | 0 | 0 | 0 | 0 | 0 | 0 | 1 (16.7) | 1 (50) | 0 | 0 |
| – Atrioventricular septal defect (AVSD) | 3773 (1.6%) | 6 (3.5) | 0 | 0 | 0 | 0 | 0 | 0 | 0 | 0 | 0 | 0 | 0 | 0 | 0 | 0 | 0 | 1 (100) | 0 |
| – Tetralogy of Fallot | 2841 (1.2%) | 7 (4.0) | 0 | 0 | 0 | 0 | 0 | 1 (50) | 0 | 0 | 0 | 0 | 2 (100) | 0 | 0 | 0 | 0 | 0 | 0 |
| – Tricuspid atresia and stenosis | 547 (0.2%) | 1 (0.6) | 0 | 0 | 0 | 0 | 0 | 0 | 0 | 0 | 0 | 0 | 0 | 0 | 0 | 0 | 0 | 0 | 0 |
| – Ebstein's anomaly | 408 (0.2%) | 0 | 0 | 0 | 0 | 0 | 0 | 0 | 0 | 0 | 0 | 0 | 0 | 0 | 0 | 0 | 0 | 0 | 0 |
| – Pulmonary valve stenosis | 3743 (1.6%) | 6 (3.5) | 0 | 0 | 0 | 0 | 0 | 0 | 0 | 0 | 0 | 1 (33.3) | 0 | 0 | 0 | 0 | 0 | 0 | 0 |
| – Pulmonary valve atresia | 840 (0.4%) | 2 (1.2) | 0 | 0 | 0 | 0 | 0 | 0 | 0 | 0 | 0 | 0 | 0 | 0 | 0 | 0 | 0 | 0 | 0 |
| – Aortic valve atresia/stenosis § | 1532 (0.7%) | 1 (0.6) | 0 | 0 | 0 | 0 | 0 | 0 | 0 | 0 | 0 | 0 | 0 | 0 | 0 | 0 | 0 | 0 | 0 |
| – Mitral valve anomalies | 1215 (0.5%) | 2 (1.2) | 0 | 0 | 0 | 0 | 0 | 0 | 0 | 0 | 0 | 0 | 0 | 0 | 0 | 0 | 0 | 0 | 0 |
| – Hypoplastic left heart | 2158 (0.9%) | 3 (1.7) | 0 | 0 | 0 | 0 | 0 | 0 | 0 | 0 | 0 | 0 | 0 | 0 | 0 | 0 | 0 | 0 | 0 |
| – Hypoplastic right heart § | 349 (0.2%) | 0 | 0 | 0 | 0 | 0 | 0 | 0 | 0 | 0 | 0 | 0 | 0 | 0 | 0 | 0 | 0 | 0 | 0 |
| – Coarctation of aorta | 3045 (1.3%) | 2 (1.2) | 0 | 0 | 0 | 0 | 0 | 0 | 0 | 0 | 0 | 0 | 0 | 0 | 0 | 0 | 0 | 0 | 0 |
| – Aortic atresia/interrupted aortic arch | 293 (0.1%) | 0 | 0 | 0 | 0 | 0 | 0 | 0 | 0 | 0 | 0 | 0 | 0 | 0 | 0 | 0 | 0 | 0 | 0 |
| – Total anomalous pulm venous return | 443 (0.2%) | 0 | 0 | 0 | 0 | 0 | 0 | 0 | 0 | 0 | 0 | 0 | 0 | 0 | 0 | 0 | 0 | 0 | 0 |
| – PDA as only CHD in term infants  (>=37 weeks) | 3329 (1.4%) | 2 (1.2) | 0 | 0 | 0 | 0 | 1 (33.3) | 0 | 0 | 0 | 0 | 0 | 0 | 0 | 0 | 1 (16.7) | 0 | 0 | 0 |
| Respiratory | 2580 (1.1%) | 2 (1.2) | 0 | 0 | 0 | 0 | 0 | 0 | 0 | 0 | 1 (33.3) | 0 | 0 | 0 | 0 | 0 | 0 | 0 | 0 |
| – Choanal atresia | 701 (0.3%) | 0 | 0 | 0 | 0 | 0 | 0 | 0 | 0 | 0 | 0 | 0 | 0 | 0 | 0 | 0 | 0 | 0 | 0 |
| – Cystic adenomatous malf of lung § | 520 (0.2%) | 1 (0.6) | 0 | 0 | 0 | 0 | 0 | 0 | 0 | 0 | 0 | 0 | 0 | 0 | 0 | 0 | 0 | 0 | 0 |
| Oro-facial clefts | 14186 (6.1%) | 1 (0.6) | 0 | 0 | 0 | 0 | 0 | 0 | 0 | 0 | 0 | 0 | 0 | 0 | 0 | 0 | 0 | 0 | 0 |
| – Cleft lip with or without palate | 8221 (3.5%) | 0 | 0 | 0 | 0 | 0 | 0 | 0 | 0 | 0 | 0 | 0 | 0 | 0 | 0 | 0 | 0 | 0 | 0 |
| – Cleft palate | 5965 (2.6%) | 1 (0.6) | 0 | 0 | 0 | 0 | 0 | 0 | 0 | 0 | 0 | 0 | 0 | 0 | 0 | 0 | 0 | 0 | 0 |
| Digestive system | 15653 (6.7%) | 17 (9.8) | 0 | 1 (50) | 0 | 0 | 0 | 2 (100) | 0 | 0 | 0 | 0 | 0 | 0 | 0 | 1 (16.7) | 1 (50) | 0 | 0 |
| – Oesophageal atresia with  or without tracheo-oesophageal fistula | 2166 (0.9%) | 5 (2.9) | 0 | 1 (50) | 0 | 0 | 0 | 1 (50) | 0 | 0 | 0 | 0 | 0 | 0 | 0 | 0 | 1 (50) | 0 | 0 |
| – Duodenal atresia or stenosis | 1041 (0.5%) | 0 | 0 | 0 | 0 | 0 | 0 | 0 | 0 | 0 | 0 | 0 | 0 | 0 | 0 | 0 | 0 | 0 | 0 |
| – Atresia or stenosis of other parts  of small intestine | 616 (0.3%) | 2 (1.2) | 0 | 0 | 0 | 0 | 0 | 0 | 0 | 0 | 0 | 0 | 0 | 0 | 0 | 0 | 0 | 0 | 0 |
| – Ano-rectal atresia and stenosis | 2714 (1.2%) | 1 (0.6) | 0 | 0 | 0 | 0 | 0 | 0 | 0 | 0 | 0 | 0 | 0 | 0 | 0 | 0 | 0 | 0 | 0 |
| – Hirschsprung's disease | 997 (0.4%) | 0 | 0 | 0 | 0 | 0 | 0 | 0 | 0 | 0 | 0 | 0 | 0 | 0 | 0 | 0 | 0 | 0 | 0 |
| – Atresia of bile ducts | 290 (0.1%) | 1 (0.6) | 0 | 0 | 0 | 0 | 0 | 1 (50) | 0 | 0 | 0 | 0 | 0 | 0 | 0 | 0 | 0 | 0 | 0 |
| – Annular pancreas | 197 (0.1%) | 1 (0.6) | 0 | 0 | 0 | 0 | 0 | 0 | 0 | 0 | 0 | 0 | 0 | 0 | 0 | 0 | 0 | 0 | 0 |
| – Diaphragmatic hernia | 2099 (0.9%) | 1 (0.6) | 0 | 0 | 0 | 0 | 0 | 0 | 0 | 0 | 0 | 0 | 0 | 0 | 0 | 0 | 0 | 0 | 0 |
| Abdominal wall defects | 4140 (1.8%) | 4 (2.3) | 0 | 0 | 0 | 0 | 1 (33.3) | 0 | 0 | 0 | 0 | 1 (33.3) | 0 | 0 | 0 | 0 | 0 | 0 | 1 (100) |
| – Gastroschisis | 1668 (0.7%) | 2 (1.2) | 0 | 0 | 0 | 0 | 0 | 0 | 0 | 0 | 0 | 0 | 0 | 0 | 0 | 0 | 0 | 0 | 0 |
| – Omphalocele | 2206 (1.0%) | 2 (1.2) | 0 | 0 | 0 | 0 | 0 | 0 | 0 | 0 | 0 | 0 | 0 | 0 | 0 | 0 | 0 | 0 | 0 |
| Urinary | 31404 (13.5%) | 19 (11.0) | 0 | 0 | 0 | 0 | 0 | 0 | 0 | 1 (25) | 0 | 0 | 0 | 0 | 0 | 2 (33.3) | 1 (50) | 0 | 0 |
| – Bilateral renal agenesis including  Potter syndrome | 848 (0.4%) | 0 | 0 | 0 | 0 | 0 | 0 | 0 | 0 | 0 | 0 | 0 | 0 | 0 | 0 | 0 | 0 | 0 | 0 |
| – Multicystic renal dysplasia | 2911 (1.3%) | 2 (1.2) | 0 | 0 | 0 | 0 | 0 | 0 | 0 | 0 | 0 | 0 | 0 | 0 | 0 | 1 (16.7) | 0 | 0 | 0 |
| – Congenital hydronephrosis | 10700 (4.6%) | 6 (3.5) | 0 | 0 | 0 | 0 | 1 (33.3) | 0 | 0 | 0 | 0 | 0 | 0 | 0 | 0 | 0 | 1 (50) | 0 | 0 |
| – Bladder exstrophy and/or epispadia | 520 (0.2%) | 0 | 0 | 0 | 0 | 0 | 0 | 0 | 0 | 0 | 0 | 0 | 0 | 0 | 0 | 0 | 0 | 0 | 0 |
| – Posterior urethral valve and/or prune  belly | 1289 (0.6%) | 1 (0.6) | 0 | 0 | 0 | 0 | 0 | 0 | 0 | 0 | 0 | 0 | 0 | 0 | 0 | 0 | 0 | 0 | 0 |
| Genital | 19853 (8.5%) | 12 (6.9) | 1 (16.7) | 0 | 0 | 0 | 0 | 0 | 0 | 0 | 2 (66.7) | 0 | 0 | 0 | 0 | 1 (16.7) | 0 | 0 | 0 |
| – Hypospadias | 16523 (7.1%) | 8 (4.6) | 1 (16.7) | 0 | 0 | 0 | 0 | 0 | 0 | 0 | 2 (66.7) | 0 | 0 | 0 | 0 | 0 | 0 | 0 | 0 |
| – Indeterminate sex | 483 (0.2%) | 0 | 0 | 0 | 0 | 0 | 0 | 0 | 0 | 0 | 0 | 0 | 0 | 0 | 0 | 0 | 0 | 0 | 0 |
| Limb | 39127 (16.8%) | 32 (18.5) | 2 (33.3) | 2 (100) | 0 | 1 (100) | 0 | 1 (50) | 0 | 0 | 0 | 0 | 0 | 0 | 1 (100) | 2 (33.3) | 1 (50) | 0 | 0 |
| – Limb reduction defects | 5319 (2.3%) | 4 (2.3) | 0 | 0 | 0 | 0 | 0 | 0 | 0 | 0 | 0 | 0 | 0 | 0 | 0 | 0 | 0 | 0 | 0 |
| – Club foot - talipes equinovarus | 9723 (4.2%) | 4 (2.3) | 0 | 1 (50) | 0 | 0 | 0 | 0 | 0 | 0 | 0 | 0 | 0 | 0 | 0 | 0 | 1 (50) | 0 | 0 |
| – Hip dislocation and/or dysplasia | 6619 (2.9%) | 4 (2.3) | 0 | 0 | 0 | 0 | 0 | 0 | 0 | 0 | 0 | 0 | 0 | 0 | 0 | 1 (16.7) | 0 | 0 | 0 |
| – Polydactyly | 10201 (4.4%) | 14 (8.1) | 0 | 0 | 0 | 1 (100) | 0 | 0 | 0 | 0 | 0 | 0 | 0 | 0 | 1 (100) | 1 (16.7) | 0 | 0 | 0 |
| – Syndactyly | 5279 (2.3%) | 4 (2.3) | 2 (33.3) | 0 | 0 | 1 (100) | 0 | 0 | 0 | 0 | 0 | 1 (33.3) | 0 | 0 | 0 | 0 | 0 | 0 | 0 |
| Other anomalies/syndromes |  |  | 2 (33.3) | 0 | 1 (50) | 0 | 0 | 0 | 0 | 1 (25) | 0 | 0 | 0 | 0 | 0 | 0 | 0 | 0 | 0 |
| – Skeletal dysplasias § | 1320 (0.6%) | 0 | 0 | 0 | 0 | 0 | 0 | 0 | 0 | 0 | 0 | 0 | 0 | 0 | 0 | 0 | 0 | 0 | 0 |
| – Craniosynostosis | 2330 (1.0%) | 0 | 0 | 0 | 0 | 0 | 0 | 0 | 0 | 0 | 0 | 0 | 0 | 0 | 0 | 0 | 0 | 0 | 0 |
| – Congenital constriction bands/ amniotic band | 422 (0.2%) | 1 (0.6) | 0 | 0 | 0 | 0 | 0 | 0 | 0 | 0 | 0 | 0 | 0 | 0 | 0 | 0 | 0 | 0 | 0 |
| – Situs inversus | 615 (0.3%) | 1 (0.6) | 0 | 0 | 0 | 0 | 0 | 0 | 0 | 0 | 0 | 0 | 0 | 0 | 0 | 0 | 0 | 0 | 0 |
| – Conjoined twins | 100 (0.04%) | 0 | 0 | 0 | 0 | 0 | 0 | 0 | 0 | 0 | 0 | 0 | 0 | 0 | 0 | 0 | 0 | 0 | 0 |
| – Congenital skin disorders | 1912 (0.8%) | 0 | 0 | 0 | 0 | 0 | 0 | 0 | 0 | 0 | 0 | 0 | 0 | 0 | 0 | 0 | 0 | 0 | 0 |
| – VATER/VACTERL | 309 (0.1%) | 0 | 0 | 0 | 0 | 0 | 0 | 0 | 0 | 0 | 0 | 0 | 0 | 0 | 0 | 0 | 0 | 0 | 0 |
| – Vascular disruption anomalies § | 5945 (2.6%) | 6 (3.5) | 1 (16.7) | 0 | 0 | 0 | 0 | 0 | 0 | 0 | 0 | 0 | 0 | 0 | 0 | 0 | 0 | 0 | 0 |
| – Lateral anomalies § | 1510 (0.7%) | 3 (1.7) | 0 | 0 | 0 | 0 | 0 | 0 | 0 | 0 | 0 | 0 | 0 | 0 | 0 | 0 | 0 | 0 | 0 |
| – Teratogenic syndromes with  malformations § | 990 (0.4%) | 4 (2.3) | 0 | 0 | 0 | 0 | 0 | 0 | 0 | 0 | 0 | 0 | 0 | 0 | 0 | 0 | 0 | 0 | 0 |
| – Fetal alcohol syndrome | 365 (0.2%) | 2 (1.2) | 0 | 0 | 0 | 0 | 0 | 0 | 0 | 0 | 0 | 0 | 0 | 0 | 0 | 0 | 0 | 0 | 0 |
| – Valproate syndrome § | 58 (0.02%) | 0 | 0 | 0 | 0 | 0 | 0 | 0 | 0 | 0 | 0 | 0 | 0 | 0 | 0 | 0 | 0 | 0 | 0 |
| – Maternal infections resulting  in malformations | 534 (0.2%) | 2 (1.2) | 0 | 0 | 0 | 0 | 0 | 0 | 0 | 0 | 0 | 0 | 0 | 0 | 0 | 0 | 0 | 0 | 0 |
| – Genetic syndromes + microdeletions | 4616 (2.0%) | 5 (2.9) | 1 (16.7) | 0 | 1 (50) | 0 | 0 | 0 | 0 | 0 | 0 | 0 | 0 | 0 | 0 | 0 | 0 | 0 | 0 |
| Chromosomal | 28454 (12.2%) | 31 (17.9) | 1 (16.7) | 0 | 0 | 0 | 1 (33.3) | 2 (66.7) | 0 | 1 (25) | 0 | 0 | 0 | 0 | 0 | 0 | 0 | 1 (100) | 0 |
| – Down Syndrome | 17003 (7.3%) | 17 (9.8) | 0 | 0 | 0 | 0 | 0 | 0 | 0 | 1 (25) | 0 | 0 | 0 | 0 | 0 | 0 | 0 | 0 | 0 |
| – Patau syndrome/trisomy 13 | 1223 (0.5%) | 2 (1.2) | 0 | 0 | 0 | 0 | 1 (33.3) | 0 | 0 | 0 | 0 | 0 | 0 | 0 | 0 | 0 | 0 | 0 | 0 |
| – Edward syndrome/trisomy 18 | 3449 (1.5%) | 12 (6.9) | 1 (16.7) | 0 | 0 | 0 | 0 | 1 (50) | 0 | 0 | 0 | 1 (33.3) | 0 | 0 | 0 | 0 | 0 | 0 | 0 |
| – Turner syndrome | 1569 (0.7%) | 0 | 0 | 0 | 0 | 0 | 0 | 0 | 0 | 0 | 0 | 0 | 0 | 0 | 0 | 0 | 0 | 0 | 0 |
| – Klinefelter syndrome | 646 (0.3%) | 1 (0.6) | 0 | 0 | 0 | 0 | 0 | 0 | 0 | 0 | 0 | 0 | 0 | 0 | 0 | 0 | 0 | 0 | 0 |

D4T: Stavudine; APV: Amprenavir; ARV: Antiretroviral; BTG: Bictegravir; DDI: Didanosine; DDC: Zalcitabine; DTG: Dolutegravir; EFV: Efavirenz; ETR: Etravirine; EVG: Elvitegravir; FPV: Fosamprenavir; IDV: Indinavir; NFV: Nelfinavir; RPV: Rilpivirine; RTG: Raltegravir; RTV: Ritonavir only; SQV: Saquinavir; TAF: Tenofovir alafenamide.

**All registries did not cover the same study period due to difference in data availability at the time of study analysis as of October 2023.*

**Additional file number 3: Description of demographic, maternal and birth data of congenital anomalies cases by exposure to any antiretroviral drug, and each specific antiretroviral drug with more than 15 cases, in 17 European registries between 1995 and 2019*.**

|  | **ARV** | | **Nucleoside reverse transcriptase inhibitors** | | | | | | | | | |  |  |
| --- | --- | --- | --- | --- | --- | --- | --- | --- | --- | --- | --- | --- | --- | --- |
|  |  | | **TDF** | | **FTC** | | **3TC** | | **ZDV** | | **ABC** | |  |  |
|  | 173 | % | 65 | % | 54 | % | 51 | % | 49 | % | 21 | % |  |  |
| **Demographic data** |  |  |  |  |  |  |  |  |  |  |  |  |  |  |
| **Registries (n=17)** |  |  |  |  |  |  |  |  |  |  |  |  |  |  |
| **France** | 87 | 50.3% | 49 | 28.3% | 42 | 24.3% | 16 | 9.2% | 6 | 3.5% | 14 | 8.1% |  |  |
| Paris | 66 | 38.2% | 33 | 50.8% | 31 | 57.4% | 14 | 27.5% | 5 | 10.2% | 12 | 57.1% |  |  |
| Brittany | 10 | 5.8% | 9 | 13.8% | 7 | 13.0% | 1 | 2.0% | 1 | 2.0% | 1 | 4.8% |  |  |
| Haute-Garonne | 6 | 3.5% | 5 | 7.7% | 2 | 3.7% | 1 | 2.0% | 0 |  | 1 | 4.8% |  |  |
| Isle de Reunion | 4 | 2.3% | 2 | 3.1% | 2 | 3.7% | 0 |  | 0 |  | 0 |  |  |  |
| Auvergne | 1 | 0.6% | 0 |  | 0 |  | 0 |  | 0 |  | 0 |  |  |  |
| **Italy** | 24 | 13.9% |  |  |  |  | 5 | 9.8% | 13 | 26.5% |  |  |  |  |
| Tuscany | 13 | 7.5% | 0 |  | 0 |  | 4 | 7.8% | 10 | 20.4% | 0 |  |  |  |
| Emilia Romagna | 11 | 6.4% | 0 |  | 0 |  | 1 | 2.0% | 3 | 6.1% | 0 |  |  |  |
| **Spain** |  |  |  |  |  |  |  |  |  |  |  |  |  |  |
| Valencian Region | 24 | 13.9% | 5 | 7.7% | 3 | 5.6% | 10 | 19.6% | 13 | 26.5% | 2 | 9.5% |  |  |
| **Switzerland** |  |  |  |  |  |  |  |  |  |  |  |  |  |  |
| Vaud | 13 | 7.5% | 3 | 4.6% | 2 | 3.7% | 9 | 17.6% | 8 | 16.3% | 1 | 4.8% |  |  |
| **Poland** |  |  |  |  |  |  |  |  |  |  |  |  |  |  |
| Poland | 7 | 4.0% | 1 | 1.5% | 1 | 1.9% | 4 | 7.8% | 4 | 8.2% | 0 |  |  |  |
| Wielkopolska | 0 |  | 0 |  | 0 |  | 0 |  | 0 |  | 0 |  |  |  |
| **Belgium** |  |  |  |  |  |  |  |  |  |  |  |  |  |  |
| Antwerp | 7 | 4.0% | 3 | 4.6% | 2 | 3.7% | 3 | 5.9% | 3 | 6.1% | 3 | 14.3% |  |  |
| **United Kingdom** |  |  |  |  |  |  |  |  |  |  |  |  |  |  |
| Wales | 6 | 3.5% | 3 | 4.6% | 3 | 5.6% | 2 | 3.9% | 1 | 2.0% | 0 |  |  |  |
| Cork and Kerry | 0 |  | 0 |  | 0 |  | 0 |  | 0 |  | 0 |  |  |  |
| **Germany** |  |  |  |  |  |  |  |  |  |  |  |  |  |  |
| Saxony Anhalt | 2 | 1.2% | 0 |  | 1 | 1.9% | 1 | 2.0% | 1 | 2.0% | 0 |  |  |  |
| **Denmark** |  |  |  |  |  |  |  |  |  |  |  |  |  |  |
| Funen | 3 | 1.7% | 1 | 1.5% | 0 | 0.0% | 1 | 2.0% | 0 |  | 1 | 4.8% |  |  |
| **Netherlands** | 0 |  | 0 |  | 0 |  | 0 |  | 0 |  | 0 |  |  |  |
| **Birth years** |  |  |  |  |  |  |  |  |  |  |  |  |  |  |
| 1995-2010 | 70 | 40.5% | 5 | 7.7% | 3 | 5.6% | 29 | 56.9% | 28 | 57.1% | 10 | 47.6% |  |  |
| 2010-2019 | 103 | 59.5% | 60 | 92.3% | 51 | 94.4% | 22 | 43.1% | 21 | 42.9% | 11 | 52.4% |  |  |
| **Maternal data** |  |  |  |  |  |  |  |  |  |  |  |  |  |  |
| **Illness before pregnancy** |  |  |  |  |  |  |  |  |  |  |  |  |  |  |
| None | 13 | 7.5% | 3 | 4.6% | 2 | 3.7% | 4 | 7.8% | 4 | 8.2% | 0 |  |  |  |
| Certain infectious and parasitic diseases |  |  |  |  |  |  |  |  |  |  |  |  |  |  |
| Viral hepatitis |  |  |  |  |  |  |  |  |  |  |  |  |  |  |
| Hepatitis B | 14 | 8.1% | 7 | 10.8% | 5 | 9.3% | 5 | 9.8% | 1 | 2.0% | 0 |  |  |  |
| Hepatitis C | 5 | 2.9% | 1 | 1.5% | 1 | 1.9% | 3 | 5.9% | 4 | 8.2% | 1 | 4.8% |  |  |
| Hepatitis not specified | 5 | 2.9% | 3 | 4.6% | 0 |  | 1 | 2.0% | 1 | 2.0% | 0 |  |  |  |
| HIV | 107 | 61.8% | 46 | 70.8% | 45 | 83.3% | 29 | 56.9% | 26 | 53.1% | 19 | 90.5% |  |  |
| Endocrine, nutritional and metabolic diseases |  |  |  |  |  |  |  |  |  |  |  |  |  |  |
| Obesity, unspecified | 12 | 6.9% | 6 | 9.2% | 5 | 9.3% | 2 | 3.9% | 0 |  | 3 | 14.3% |  |  |
| Diseases of the circulatory system |  |  |  |  |  |  |  |  |  |  |  |  |  |  |
| Essential (primary) hypertension | 4 | 2.3% | 3 | 4.6% | 2 | 3.7% | 0 |  | 0 |  | 1 | 4.8% |  |  |
| Missing data | 32 | 18.5% | 11 | 16.9% | 7 | 13.0% | 9 | 17.6% | 13 | 26.5% | 2 | 9.5% |  |  |
| **Illness during pregnancy** |  |  |  |  |  |  |  |  |  |  |  |  |  |  |
| None | 82 | 47.4% | 38 | 58.5% | 34 | 63.0% | 17 | 33.3% | 12 | 24.5% | 13 | 61.9% |  |  |
| Certain infectious and parasitic diseases |  |  |  |  |  |  |  |  |  |  |  |  |  |  |
| HIV | 20 | 11.6% | 4 | 6.2% | 5 | 9.3% | 9 | 17.6% | 12 | 24.5% | 0 |  |  |  |
| Pregnancy, childbirth and the puerperium |  |  |  |  |  |  |  |  |  |  |  |  |  |  |
| Diabetes mellitus arising in pregnancy | 5 | 2.9% | 2 | 3.1% | 2 | 3.7% | 1 | 2.0% | 0 |  | 0 |  |  |  |
| Missing data | 35 | 20.2% | 12 | 18.5% | 8 | 14.8% | 12 | 23.5% | 15 | 30.6% | 3 | 14.3% |  |  |
| **Maternal age (years)** |  |  |  |  |  |  |  |  |  |  |  |  |  |  |
| Median (IQR) | 34.0  [29.0-38.0] | | 35.0  [30.0-38.0] | | 35.5  [30.0-39.0] | | 32.0  [27.0-35.0] | | 31.0  [27.0-37.0] | | 35.0  [29.0-40.0] | |  |  |
| 14-19 years | 1 | 0.6% | 2 | 3.1% | 2 | 3.7% | 0 |  | 0 |  | 0 |  |  |  |
| 20-29 years | 16 | 9.2% | 12 | 18.5% | 11 | 20.4% | 21 | 41.2% | 20 | 40.8% | 4 | 19.0% |  |  |
| 30-40 years | 89 | 51.4% | 44 | 67.7% | 34 | 63.0% | 27 | 52.9% | 27 | 55.1% | 16 | 76.2% |  |  |
| ≥41 years | 67 | 38.7% | 7 | 10.8% | 7 | 13.0% | 3 | 5.9% | 2 | 4.1% | 1 | 4.8% |  |  |
| **Birth data** |  |  |  |  |  |  |  |  |  |  |  |  |  |  |
| **Number of fetuses/babies per delivery** |  |  |  |  |  |  |  |  |  |  |  |  |  |  |
| 1 | 161 | 93.1% | 61 | 93.8% | 52 | 96.3% | 47 | 92.2% | 46 | 93.9% | 19 | 90.5% |  |  |
| 2 | 12 | 6.9% | 4 | 6.2% | 2 | 3.7% | 4 | 7.8% | 3 | 6.1% | 1 | 4.8% |  |  |
| **Sex** |  |  |  |  |  |  |  |  |  |  |  |  |  |  |
| Male | 87 | 50.3% | 32 | 49.2% | 27 | 50.0% | 26 | 51.0% | 29 | 59.2% | 13 | 61.9% |  |  |
| Female | 84 | 48.6% | 31 | 47.7% | 26 | 48.1% | 25 | 49.0% | 20 | 40.8% | 7 | 33.3% |  |  |
| Missing data | 2 | 1.2% | 2 | 3.1% | 1 | 1.9% | 0 |  | 0 |  | 0 |  |  |  |
| **Pregnancy outcomes** |  |  |  |  |  |  |  |  |  |  |  |  |  |  |
| Live birth | 143 | 82.7% | 48 | 73.8% | 39 | 72.2% | 45 | 95.7% | 46 | 93.9% | 16 | 84.2% |  |  |
| Preterm births (<37 weeks) | 34 | 23.8% | 11 | 22.9% | 10 | 22.2% | 11 | 23.9% | 8 | 20.0% | 3 | 18.8% |  |  |
| Low-birth-weight (<2500grs) | 46 | 32.2% | 16 | 33.3% | 14 | 31.1% | 16 | 34.8% | 13 | 32.5% | 7 | 43.8% |  |  |
| Fetal death >=20 weeks | 6 | 3.5% | 4 | 6.2% | 4 | 7.4% | 2 | 4.3% | 0 |  | 0 |  |  |  |
| TOPFA | 24 | 13.9% | 13 | 20.0% | 11 | 20.4% | 4 | 8.5% | 3 | 6.1% | 4 | 21.1% |  |  |
| **Classification of the anomalies** |  |  |  |  |  |  |  |  |  |  |  |  |  |  |
| Isolated cardiac | 53 | 31.0% | 16 | 24.6% | 14 | 25.9% | 18 | 35.3% | 21 | 42.9% | 7 | 33.3% |  |  |
| Genetic syndrome, skeletal dysplasia and monogenetic disorder | 5 | 2.9% | 0 |  | 0 |  | 2 | 3.9% | 1 | 2.0% | 1 | 4.8% |  |  |
| Chromosomal | 32 | 18.5% | 14 | 21.5% | 12 | 22.2% | 7 | 13.7% | 6 | 12.2% | 5 | 23.8% |  |  |
| Isolated other | 48 | 27.7% | 15 | 23.1% | 12 | 22.2% | 17 | 33.3% | 17 | 34.7% | 6 | 28.6% |  |  |
| Potential multiple anomalies | 15 | 8.7% | 6 | 9.2% | 6 | 11.1% | 4 | 7.8% | 2 | 4.1% | 1 | 4.8% |  |  |
| Neural tube defect isolated | 1 | 0.6% | 1 | 1.5% | 1 | 1.9% | 0 |  | 0 |  | 0 |  |  |  |
| Isolated renal | 9 | 5.2% | 6 | 9.2% | 5 | 9.3% | 2 | 3.9% | 2 | 4.1% | 0 |  |  |  |
| Teratogenic syndrome | 4 | 2.3% | 2 | 3.1% | 2 | 3.7% | 0 |  | 0 |  | 0 |  |  |  |
|  | 0 |  | 0 |  | 0 |  | 0 |  | 0 |  | 0 |  |  |  |
| Missing data | 6 | 3.5% | 5 | 7.7% | 2 | 3.7% | 1 | 2.0% |  |  | 1 | 4.8% |  |  |
| **Diagnosis** |  |  |  |  |  |  |  |  |  |  |  |  |  |  |
| At birth | 63 | 36.4% | 19 | 29.2% | 16 | 29.6% | 22 | 43.1% | 21 | 42.9% | 12 | 60.0% |  |  |
| Less than 1 week | 14 | 8.1% | 3 | 4.6% | 2 | 3.7% | 5 | 9.8% | 7 | 14.3% | 1 | 5.0% |  |  |
| 1-4 weeks | 4 | 2.3% | 2 | 3.1% | 2 | 3.7% | 1 | 2.0% | 1 | 2.0% | 0 |  |  |  |
| 1-12 months | 5 | 2.9% | 1 | 1.5% | 1 | 1.9% | 2 | 3.9% | 2 | 4.1% | 1 | 5.0% |  |  |
| >12 months | 2 | 1.2% | 0 |  | 0 |  | 1 | 2.0% | 1 | 2.0% | 0 |  |  |  |
| Prenatal diagnosis in **live** foetuses | 79 | 45.7% | 39 | 60.0% | 33 | 61.1% | 18 | 35.3% | 15 | 30.6% | 6 | 30.0% |  |  |
| Not known | 5 | 2.9% | 1 | 1.5% | 0 |  | 1 | 2.0% | 1 | 2.0% | 1 | 5.0% |  |  |
| Postnatal, age not known | 1 | 0.6% | 0 |  | 0 |  | 1 | 2.0% | 1 | 2.0% | 0 |  |  |  |
| **Gestational age for prenatal diagnosis** | 83 |  | 42 |  | 35 |  | 18 |  | 15 |  | 7 |  |  |  |
| 0-22 weeks | 41 | 49.4% | 20 | 47.6% | 17 | 48.6% | 8 | 44.4% | 7 | 46.7% | 4 | 57.1% |  |  |
| 23-27 weeks | 13 | 15.7% | 8 | 19.0% | 8 | 22.9% | 3 | 16.7% | 2 | 13.3% | 1 | 14.3% |  |  |
| 28-31 weeks | 6 | 7.2% | 1 | 2.4% | 1 | 2.9% | 3 | 16.7% | 2 | 13.3% | 0 |  |  |  |
| 32-36 weeks | 9 | 10.8% | 5 | 11.9% | 4 | 11.4% | 3 | 16.7% | 2 | 13.3% | 0 |  |  |  |
| Missing data | 14 | 16.9% | 8 | 19.0% | 5 | 14.3% | 1 | 5.6% | 2 | 13.3% | 2 | 28.6% |  |  |

**Additional file number 3 continued: Description of demographic, maternal and birth data of congenital anomalies cases by exposure to any antiretroviral drug, and each specific antiretroviral drug with more than 15 cases, in 17 European registries between 1995 and 2019*.**

|  | **ARV** | | **Protease inhibitors** | | | | | | **Non-nucleoside reverse transcriptase inhibitors** | |
| --- | --- | --- | --- | --- | --- | --- | --- | --- | --- | --- |
|  |  |  | **LPV** | | **ATV** | | **DRV** | | **NVP** | |
|  | 173 | % | 48 | % | 17 | % | 15 | % | 20 | % |
| **Demographic data** |  |  |  |  |  |  |  |  |  |  |
| **Registries (n=17)** |  |  |  |  |  |  |  |  |  |  |
| **France** | 87 | 50.3% | 21 | 12.1% | 15 | 8.7% | 13 | 86.7% | 15 | 8.7% |
| Paris | 66 | 38.2% | 19 | 39.6% | 11 | 64.7% | 10 | 66.7% | 12 | 60.0% |
| Brittany | 10 | 5.8% | 2 | 4.2% | 2 | 11.8% | 1 | 6.7% | 3 | 15.0% |
| Haute-Garonne | 6 | 3.5% | 0 |  | 1 | 5.9% | 0 |  | 0 |  |
| Isle de Reunion | 4 | 2.3% | 0 |  | 1 | 5.9% | 1 | 6.7% | 0 |  |
| Auvergne | 1 | 0.6% | 0 |  | 0 |  | 1 | 6.7% | 0 |  |
| **Italy** | 24 | 13.9% | 7 | 14.6% |  |  |  |  | 1 | 5.0% |
| Tuscany | 13 | 7.5% | 4 | 8.3% | 0 |  | 0 |  | 0 |  |
| Emilia Romagna | 11 | 6.4% | 3 | 6.3% | 0 |  | 0 |  | 1 | 5.0% |
| **Spain** |  |  |  |  |  |  | 1 | 6.7% |  |  |
| Valencian Region | 24 | 13.9% | 10 | 20.8% | 0 |  | 1 | 6.7% | 1 | 5.0% |
| **Switzerland** |  |  |  |  |  |  |  |  |  |  |
| Vaud | 13 | 7.5% | 7 | 14.6% | 0 |  | 0 |  | 0 |  |
| **Poland** |  |  |  |  |  |  |  |  |  |  |
| Poland | 7 | 4.0% | 0 |  | 0 |  | 0 |  | 0 |  |
| Wielkopolska | 0 |  | 0 |  | 0 |  | 0 |  | 0 |  |
| **Belgium** |  |  |  |  |  |  |  |  |  |  |
| Antwerp | 7 | 4.0% | 2 | 4.2% | 1 | 5.9% | 0 |  | 2 | 10.0% |
| **United Kingdom** |  |  |  |  |  |  | 1 | 6.7% |  |  |
| Wales | 6 | 3.5% | 0 |  | 0 |  | 1 | 6.7% | 0 |  |
| Cork and Kerry | 0 |  | 0 |  | 0 |  | 0 |  | 0 |  |
| **Germany** |  |  |  |  |  |  |  |  |  |  |
| Saxony Anhalt | 2 | 1.2% | 1 | 2.1% | 0 |  | 0 |  | 0 |  |
| **Denmark** |  |  |  |  |  |  |  |  |  |  |
| Funen | 3 | 1.7% | 1 | 2.1% | 1 | 5.9% | 0 |  | 0 |  |
| **Netherlands** | 0 |  | 0 |  | 0 |  | 0 |  | 0 |  |
| **Birth years** |  |  |  |  |  |  |  |  |  |  |
| 1995-2010 | 70 | 40.5% | 22 | 45.8% | 5 | 29.4% | 0 |  | 9 | 45.0% |
| 2010-2019 | 103 | 59.5% | 26 | 54.2% | 12 | 70.6% | 15 | 100% | 11 | 55.0% |
| **Maternal data** |  |  |  |  |  |  |  |  |  |  |
| **Illness before pregnancy** |  |  |  |  |  |  |  |  |  |  |
| None | 13 | 7.5% | 3 | 6.3% | 0 |  | 0 |  | 1 | 5.0% |
| Certain infectious and parasitic diseases |  |  |  |  |  |  |  |  |  |  |
| Viral hepatitis |  |  |  |  |  |  |  |  |  |  |
| Hepatitis B | 14 | 8.1% | 6 | 12.5% | 1 | 5.9% | 2 | 13.3% | 0 |  |
| Hepatitis C | 5 | 2.9% | 1 | 2.1% | 0 |  | 1 | 6.7% | 1 | 5.0% |
| Hepatitis not specified | 5 | 2.9% | 0 |  | 0 |  | 0 |  | 0 |  |
| HIV | 107 | 61.8% | 35 | 72.9% | 16 | 94.1% | 13 | 86.7% | 14 | 70.0% |
| Endocrine, nutritional and metabolic diseases |  |  |  |  |  |  |  |  |  |  |
| Obesity, unspecified | 12 | 6.9% | 4 | 8.3% | 2 | 11.8% | 1 | 6.7% | 1 | 5.0% |
| Diseases of the circulatory system |  |  |  |  |  |  |  |  |  |  |
| Essential (primary) hypertension | 4 | 2.3% | 0 |  | 1 | 5.9% | 0 |  | 1 | 5.0% |
| Missing data | 32 | 18.5% | 5 | 10.4% | 1 | 5.9% | 2 | 13.3% | 3 | 15.0% |
| **Illness during pregnancy** |  |  |  |  |  |  |  |  |  |  |
| None | 82 | 47.4% | 20 | 41.7% | 10 | 58.8% | 0 |  | 13 | 65.0% |
| Certain infectious and parasitic diseases |  |  |  |  |  |  |  |  |  |  |
| HIV | 20 | 11.6% | 8 | 16.7% | 2 | 11.8% | 0 |  | 1 | 5.0% |
| Pregnancy, childbirth and the puerperium |  |  |  |  |  |  |  |  |  |  |
| Diabetes mellitus arising in pregnancy | 5 | 2.9% | 1 | 2.1% | 1 | 5.9% | 0 |  | 0 |  |
| Missing data | 35 | 20.2% | 7 | 14.6% | 1 | 5.9% | 2 | 13.3% | 4 | 20.0% |
| **Maternal age (years)** |  |  |  |  |  |  |  |  |  |  |
| Median (IQR) | 35.0  [30.0-38.0] | | 33.0  [29.5-37.0] | | 35.0  [32.0-38.0] | | 33.0  [28.0-39.0] | | 35.0  [29.0-40.0] | |
| 14-19 years | 1 | 0.6% | 0 |  | 0 |  | 1 | 6.7% | 0 |  |
| 20-29 years | 16 | 9.2% | 12 | 25.0% | 1 | 5.9% | 4 | 26.7% | 5 | 25.0% |
| 30-40 years | 89 | 51.4% | 33 | 68.8% | 15 | 88.2% | 8 | 53.3% | 11 | 55.0% |
| ≥41 years | 67 | 38.7% | 3 | 6.3% | 1 | 5.9% | 2 | 13.3% | 4 | 20.0% |
| **Birth data** |  |  |  |  |  |  |  |  |  |  |
| **Number of fetuses/babies per delivery** |  |  |  |  |  |  |  |  |  |  |
| 1 | 161 | 93.1% | 46 | 95.8% | 16 | 94.1% | 14 | 93.3% | 20 | 100.0% |
| 2 | 12 | 6.9% | 3 | 6.3% | 1 | 5.9% | 1 | 6.7% | 1 | 5.0% |
| **Sex** |  |  |  |  |  |  |  |  |  |  |
| Male | 87 | 50.3% | 25 | 52.1% | 7 | 41.2% | 9 | 60.0% | 11 | 55.0% |
| Female | 84 | 48.6% | 23 | 47.9% | 10 | 58.8% | 6 | 40.0% | 10 | 50.0% |
| Missing data | 2 | 1.2% | 0 |  | 0 |  | 0 |  | 0 |  |
| **Pregnancy outcomes** |  |  |  |  |  |  |  |  |  |  |
| Live birth | 143 | 82.7% | 40 | 83.3% | 14 | 82.4% | 9 | 60.0% | 17 | 81.0% |
| Preterm births (<37 weeks) | 34 | 23.8% | 8 | 20.0% | 4 | 28.6% | 4 | 26.7% | 1 | 5.9% |
| Low-birth-weight (<2500grs) | 46 | 32.2% | 13 | 32.5% | 6 | 42.9% | 2 | 13.3% | 4 | 23.5% |
| Fetal death >=20 weeks | 6 | 3.5% | 2 | 4.2% | 3 | 17.6% | 1 | 6.7% | 2 | 9.5% |
| TOPFA | 24 | 13.9% | 6 | 12.5% | 0 |  | 5 | 33.3% | 2 | 9.5% |
| **Classification of the anomalies** |  |  |  |  |  |  |  |  |  |  |
| Isolated cardiac | 53 | 31.0% | 15 | 31.3% | 5 | 29.4% | 4 | 26.7% | 7 | 35.0% |
| Genetic syndrome, skeletal dysplasia and monogenetic disorder | 5 | 2.9% | 0 |  | 1 | 5.9% | 0 |  | 4 | 20.0% |
| Chromosomal | 32 | 18.5% | 11 | 22.9% | 3 | 17.6% | 4 | 26.7% | 0 |  |
| Isolated other | 48 | 27.7% | 14 | 29.2% | 3 | 17.6% | 4 | 26.7% | 6 | 30.0% |
| Potential multiple anomalies | 15 | 8.7% | 6 | 12.5% | 0 |  | 1 | 6.7% | 3 | 15.0% |
| Neural tube defect isolated | 1 | 0.6% | 0 |  | 0 |  | 1 | 6.7% | 0 |  |
| Isolated renal | 9 | 5.2% | 2 | 4.2% | 2 | 11.8% | 0 |  | 0 |  |
| Teratogenic syndrome | 4 | 2.3% | 0 |  | 2 | 11.8% | 1 | 6.7% | 0 |  |
|  | 0 |  | 0 |  | 0 |  | 0 |  | 0 |  |
| Missing data | 2 | 1.2% |  |  | 1 | 5.9% | 0 |  |  |  |
| **Diagnosis** |  |  |  |  |  |  |  |  |  |  |
| At birth | 63 | 36.4% | 20 | 41.7% | 7 | 41.2% | 6 | 40.0% | 6 | 28.6% |
| Less than 1 week | 14 | 8.1% | 4 | 8.3% | 0 |  | 0 |  | 1 | 4.8% |
| 1-4 weeks | 4 | 2.3% | 1 | 2.1% | 2 | 11.8% | 0 |  | 1 | 4.8% |
| 1-12 months | 5 | 2.9% | 1 | 2.1% | 1 | 5.9% | 0 |  | 0 |  |
| >12 months | 2 | 1.2% | 0 |  | 0 |  | 0 |  | 0 |  |
| Prenatal diagnosis in **live** foetuses | 79 | 45.7% | 21 | 43.8% | 7 | 41.2% | 9 | 60.0% | 12 | 57.1% |
| Not known | 5 | 2.9% | 0 |  | 0 |  | 0 |  | 0 |  |
| Postnatal, age not known | 1 | 0.6% | 1 | 2.1% | 0 |  | 0 |  | 0 |  |
| **Gestational age for prenatal diagnosis** | 83 |  | 21 |  | 9 |  |  |  | 12 |  |
| 0-22 weeks | 41 | 49.4% | 15 | 71.4% | 2 | 22.2% | 7 | 46.7% | 5 | 41.7% |
| 23-27 weeks | 13 | 15.7% | 2 | 9.5% | 1 | 11.1% | 1 | 6.7% | 3 | 25.0% |
| 28-31 weeks | 6 | 7.2% | 2 | 9.5% | 1 | 11.1% | 0 |  | 0 |  |
| 32-36 weeks | 9 | 10.8% | 1 | 4.8% | 2 | 22.2% | 1 | 6.7% | 3 | 25.0% |
| Missing data | 14 | 16.9% | 1 | 4.8% | 3 | 33.3% | 6 | 40.0% | 1 | 8.3% |

*ABC: abacavir; ARV: antiretroviral drug; ATV: atazanavir; FTC: emtricitabine; IQR: interquartile; LPV: lopinavir; NVP: nevirapine; TDF: tenofovir; TOPFA: termination of pregnancy for fetal anomaly; ZDV: zidovudine; 3TC: lamivudine.*

**All registries did not cover the same study period due to difference in data availability at the time of study analysis as of October 2023.*

**Additional file number 4: Description of congenital anomalies according to EUROCAT subgroup and by specific antiretroviral drug (n>15), after exposure to antiretroviral drugs, in 17 European registries between 1995 and 2019*.**

|  | **Unexposed to ARV** | **ARV** | | **Nucleoside reverse transcriptase inhibitors** | | | | | | | | | |
| --- | --- | --- | --- | --- | --- | --- | --- | --- | --- | --- | --- | --- | --- |
|  |  |  | | **TDF** | | **FTC** | | **3TC** | | **ZDV** | | **ABC** | |
| **Anomaly group** | 232418 | 173 | % | 65 | % | 54 | % | 51 | % | 49 | % | 21 | % |
| **Nervous system** | 20841 (9.0%) | 18 | 10.4% | 10 | 15.4% | 9 | 16.7% | 5 | 9.8% | 2 | 4.1% | 2 | 9.5% |
| – Neural Tube Defects | 7284 (3.1%) | 1 | 0.6% | 1 | 1.5% | 1 | 1.9% | 0 |  | 0 |  | 0 |  |
| – – Anencephalus and similar | 2078 (0.9%) | 0 |  | 0 |  | 0 |  | 0 |  | 0 |  | 0 |  |
| – – Encephalocele | 939 (0.4%) | 0 |  | 0 |  | 0 |  | 0 |  | 0 |  | 0 |  |
| – – Spina Bifida | 4267 (1.8%) | 1 | 0.6% | 1 | 1.5% | 1 | 1.9% | 0 |  | 0 |  | 0 |  |
| – Microcephaly | 2580 (1.1%) | 3 | 1.7% | 0 |  | 0 |  | 1 | 2.0% | 0 |  | 1 | 4.8% |
| – Hydrocephalus | 4463 (1.9%) | 3 | 1.7% | 1 | 1.5% | 1 | 1.9% | 2 | 3.9% | 2 | 4.1% | 1 | 4.8% |
| – Arhinencephaly/holoprosencephaly | 1000 (0.4%) | 0 |  | 0 |  | 0 |  | 0 |  | 0 |  | 0 |  |
| **Eye** | 4094 (1.8%) | 0 |  | 0 |  | 0 |  | 0 |  | 0 |  | 0 |  |
| – Anophthalmos/micropthalmos | 945 (0.4%) | 0 |  | 0 |  | 0 |  | 0 |  | 0 |  | 0 |  |
| – – Anophthalmos | 209 (0.1%) | 0 |  | 0 |  | 0 |  | 0 |  | 0 |  | 0 |  |
| – Congenital cataract | 1185 (0.5%) | 0 |  | 0 |  | 0 |  | 0 |  | 0 |  | 0 |  |
| – Congenital glaucoma | 304 (0.1%) | 0 |  | 0 |  | 0 |  | 0 |  | 0 |  | 0 |  |
| **Ear, face and neck** | 2703 (1.2%) | 2 | 1.2% | 0 |  | 0 |  | 0 |  | 0 |  | 0 |  |
| – Anotia | 335 (0.1%) | 0 |  | 0 |  | 0 |  | 0 |  | 0 |  | 0 |  |
| **Congenital heart defects** | 79676 (34.3%) | 73 | 42.2% | 25 | 38.5% | 22 | 40.7% | 24 | 47.1% | 25 | 51.0% | 11 | 52.4% |
| – Severe CHD § | 19866 (8.6%) | 26 | 15.0% | 9 | 13.8% | 9 | 16.7% | 7 | 13.7% | 6 | 12.2% | 4 | 19.0% |
| – Common arterial truncus | 649 (0.3%) | 2 | 1.2% | 1 | 1.5% | 1 | 1.9% | 1 | 2.0% | 0 |  | 1 | 4.8% |
| – Double outlet right ventricle § | 928 (0.4%) | 1 | 0.6% | 0 |  | 0 |  | 1 | 2.0% | 0 |  | 0 |  |
| – Transposition of great vessels | 2881 (1.2%) | 2 | 1.2% | 0 |  | 1 | 1.9% | 0 |  | 0 |  | 0 |  |
| – Single ventricle | 627 (0.3%) | 0 |  | 0 |  | 0 |  | 0 |  | 0 |  | 0 |  |
| – Ventricular septal defect (VSD) | 38634 (16.6%) | 29 | 16.8% | 8 | 12.3% | 8 | 14.8% | 10 | 19.6% | 8 | 16.3% | 5 | 23.8% |
| – Atrial septal defect (ASD) | 19076 (8.2%) | 12 | 6.9% | 5 | 7.7% | 5 | 9.3% | 4 | 7.8% | 6 | 12.2% | 0 |  |
| – Atrioventricular septal defect (AVSD) | 3773 (1.6%) | 6 | 3.5% | 2 | 3.1% | 2 | 3.7% | 2 | 3.9% | 2 | 4.1% | 1 | 4.8% |
| – Tetralogy of Fallot | 2841 (1.2%) | 7 | 4.0% | 3 | 4.6% | 3 | 5.6% | 2 | 3.9% | 2 | 4.1% | 0 |  |
| – Tricuspid atresia and stenosis | 547 (0.2%) | 1 | 0.6% | 0 |  | 0 |  | 0 |  | 0 |  | 0 |  |
| – Ebstein's anomaly | 408 (0.2%) | 0 |  | 0 |  | 0 |  | 0 |  | 0 |  | 0 |  |
| – Pulmonary valve stenosis | 3743 (1.6%) | 6 | 3.5% | 2 | 3.1% | 2 | 3.7% | 1 | 2.0% | 2 | 4.1% | 0 |  |
| – Pulmonary valve atresia | 840 (0.4%) | 2 | 1.2% | 1 | 1.5% | 0 |  | 1 | 2.0% | 0 |  | 2 | 9.5% |
| – Aortic valve atresia/stenosis § | 1532 (0.7%) | 1 | 0.6% | 0 |  | 0 |  | 1 | 2.0% | 1 | 2.0% | 0 |  |
| – Mitral valve anomalies | 1215 (0.5%) | 2 | 1.2% | 1 | 1.5% | 1 | 1.9% | 0 |  | 0 |  | 0 |  |
| – Hypoplastic left heart | 2158 (0.9%) | 3 | 1.7% | 2 | 3.1% | 2 | 3.7% | 0 |  | 1 | 2.0% | 0 |  |
| – Hypoplastic right heart § | 349 (0.2%) | 0 |  | 0 |  | 0 |  | 0 |  | 0 |  | 0 |  |
| – Coarctation of aorta | 3045 (1.3%) | 2 | 1.2% | 2 | 3.1% | 2 | 3.7% | 0 |  | 0 |  | 0 |  |
| – Aortic atresia/interrupted aortic arch | 293 (0.1%) | 0 |  | 0 |  | 0 |  | 0 |  | 0 |  | 0 |  |
| – Total anomalous pulm venous return | 443 (0.2%) | 0 |  | 0 |  | 0 |  | 0 |  | 0 |  | 0 |  |
| – PDA as only CHD in term infants (>=37 weeks) | 3329 (1.4%) | 2 | 1.2% | 0 |  | 0 |  | 0 |  | 1 | 2.0% | 0 |  |
| **Respiratory** | 2580 (1.1%) | 2 | 1.2% | 0 |  | 0 |  | 1 | 2.0% | 1 | 2.0% | 0 |  |
| – Choanal atresia | 701 (0.3%) | 0 |  | 0 |  | 0 |  | 0 |  | 0 |  | 0 |  |
| – Cystic adenomatous malf of lung § | 520 (0.2%) | 1 | 0.6% | 0 |  | 0 |  | 0 |  | 0 |  | 0 |  |
| **Oro-facial clefts** | 14186 (6.1%) | 1 | 0.6% | 0 |  | 0 |  | 0 |  | 0 |  | 0 |  |
| – Cleft lip with or without palate | 8221 (3.5%) | 0 |  | 0 |  | 0 |  | 0 |  | 0 |  | 0 |  |
| – Cleft palate | 5965 (2.6%) | 1 | 0.6% | 0 |  | 0 |  | 0 |  | 0 |  | 0 |  |
| **Digestive system** | 15653 (6.7%) | 17 | 9.8% | 6 | 9.2% | 6 | 11.1% | 4 | 7.8% | 2 | 4.1% | 2 | 9.5% |
| – Oesophageal atresia with or without tracheo-oesophageal fistula | 2166 (0.9%) | 5 | 2.9% | 2 | 3.1% | 2 | 3.7% | 0 |  | 0 |  | 0 |  |
| – Duodenal atresia or stenosis | 1041 (0.5%) | 0 |  | 0 |  | 0 |  | 0 |  | 0 |  | 0 |  |
| – Atresia or stenosis of other parts of small intestine | 616 (0.3%) | 2 | 1.2% | 0 |  | 0 |  | 1 | 2.0% | 0 |  | 1 | 4.8% |
| – Ano-rectal atresia and stenosis | 2714 (1.2%) | 1 | 0.6% | 0 |  | 0 |  | 1 | 2.0% | 0 |  | 0 |  |
| – Hirschsprung's disease | 997 (0.4%) | 0 |  | 0 |  | 0 |  | 0 |  | 0 |  | 0 |  |
| – Atresia of bile ducts | 290 (0.1%) | 1 | 0.6% | 1 | 1.5% | 1 | 1.9% | 0 |  | 0 |  | 0 |  |
| – Annular pancreas | 197 (0.1%) | 1 | 0.6% | 1 | 1.5% | 1 | 1.9% | 0 |  | 0 |  | 0 |  |
| – Diaphragmatic hernia | 2099 (0.9%) | 1 | 0.6% | 1 | 1.5% | 0 | 0.0% | 0 |  | 1 | 2.0% | 0 |  |
| **Abdominal wall defects** | 4140 (1.8%) | 4 | 2.3% | 0 |  | 0 |  | 2 | 3.9% | 3 | 6.1% | 0 |  |
| – Gastroschisis | 1668 (0.7%) | 2 | 1.2% | 0 |  | 0 |  | 2 | 3.9% | 2 | 4.1% | 0 |  |
| – Omphalocele | 2206 (1.0%) | 2 | 1.2% | 0 |  | 0 |  | 0 |  | 1 | 2.0% | 0 |  |
| **Urinary** | 31404 (13.5%) | 19 | 11.0% | 10 | 15.4% | 8 | 14.8% | 6 | 11.8% | 4 | 8.2% | 2 | 9.5% |
| – Bilateral renal agenesis including Potter syndrome | 848 (0.4%) | 0 |  | 0 |  | 0 |  | 0 |  | 0 |  | 0 |  |
| – Multicystic renal dysplasia | 2911 (1.3%) | 2 | 1.2% | 2 | 3.1% | 2 | 3.7% | 0 |  | 0 |  | 0 |  |
| – Congenital hydronephrosis | 10700 (4.6%) | 6 | 3.5% | 4 | 6.2% | 2 | 3.7% | 2 | 3.9% | 2 | 4.1% | 0 |  |
| – Bladder exstrophy and/or epispadia | 520 (0.2%) | 0 |  | 0 |  | 0 |  | 0 |  | 0 |  | 0 |  |
| – Posterior urethral valve and/or prune belly | 1289 (0.6%) | 1 | 0.6% | 0 |  | 0 |  | 1 | 2.0% | 1 | 2.0% | 0 |  |
| **Genital** | 19853 (8.5%) | 12 | 6.9% | 7 | 10.8% | 6 | 11.1% | 3 | 5.9% | 4 | 8.2% | 0 |  |
| – Hypospadias | 16523 (7.1%) | 8 | 4.6% | 2 | 3.1% | 1 | 1.9% | 2 | 3.9% | 4 | 8.2% | 0 |  |
| – Indeterminate sex | 483 (0.2%) | 0 |  | 0 |  | 0 |  | 0 |  | 0 |  | 0 |  |
| **Limb** | 39127 (16.8%) | 32 | 18.5% | 13 | 20.0% | 12 | 22.2% | 7 | 13.7% | 5 | 10.2% | 4 | 19.0% |
| – Limb reduction defects | 5319 (2.3%) | 4 | 2.3% | 0 |  | 0 |  | 2 | 3.9% | 0 |  | 2 | 9.5% |
| – Club foot - talipes equinovarus | 9723 (4.2%) | 4 | 2.3% | 0 |  | 1 | 1.9% | 1 | 2.0% | 1 | 2.0% | 0 |  |
| – Hip dislocation and/or dysplasia | 6619 (2.9%) | 4 | 2.3% | 2 | 3.1% | 2 | 3.7% | 2 | 3.9% | 1 | 2.0% | 1 | 4.8% |
| – Polydactyly | 10201 (4.4%) | 14 | 8.1% | 7 | 10.8% | 5 | 9.3% | 2 | 3.9% | 2 | 4.1% | 2 | 9.5% |
| – Syndactyly | 5279 (2.3%) | 4 | 2.3% | 0 |  | 0 |  | 2 | 3.9% | 1 | 2.0% | 0 |  |
| **Other anomalies/syndromes** |  |  |  |  |  |  |  |  |  |  |  |  |  |
| – Skeletal dysplasias § | 1320 (0.6%) | 0 |  | 0 |  | 0 |  | 0 |  | 0 |  | 0 |  |
| – Craniosynostosis | 2330 (1.0%) | 0 |  | 0 |  | 0 |  | 0 |  | 0 |  | 0 |  |
| – Congenital constriction bands/amniotic band | 422 (0.2%) | 1 | 0.6% | 0 |  | 0 |  | 1 | 2.0% | 0 |  | 1 | 4.8% |
| – Situs inversus | 615 (0.3%) | 1 | 0.6% | 1 | 1.5% | 1 | 1.9% | 0 |  | 0 |  | 0 |  |
| – Conjoined twins | 100 (0.04%) | 0 |  | 0 |  | 0 |  | 0 |  | 0 |  | 0 |  |
| – Congenital skin disorders | 1912 (0.8%) | 0 |  | 0 |  | 0 |  | 0 |  | 0 |  | 0 |  |
| – VATER/VACTERL | 309 (0.1%) | 0 |  | 0 |  | 0 |  | 0 |  | 0 |  | 0 |  |
| – Vascular disruption anomalies § | 5945 (2.6%) | 6 | 3.5% | 0 |  | 0 |  | 4 | 7.8% | 2 | 4.1% | 2 | 9.5% |
| – Lateral anomalies § | 1510 (0.7%) | 3 | 1.7% | 1 | 1.5% | 1 | 1.9% | 2 | 3.9% | 1 | 2.0% | 0 |  |
| – Teratogenic syndromes with malformations § | 990 (0.4%) | 4 | 2.3% | 2 | 3.1% | 2 | 3.7% | 0 |  | 0 |  | 0 |  |
| – Fetal alcohol syndrome | 365 (0.2%) | 2 | 1.2% | 1 | 1.5% | 1 | 1.9% | 0 |  | 0 |  | 0 |  |
| – Valproate syndrome § | 58 (0.02%) | 0 |  | 0 |  | 0 |  | 0 |  | 0 |  | 0 |  |
| – Maternal infections resulting in malformations | 534 (0.2%) | 2 | 1.2% | 1 | 1.5% | 1 | 1.9% | 0 |  | 0 |  | 0 |  |
| – Genetic syndromes + microdeletions | 4616 (2.0%) | 5 | 2.9% | 0 |  | 0 |  | 2 | 3.9% | 1 | 2.0% | 1 | 4.8% |
| **Chromosomal** | 28454 (12.2%) | 31 | 17.9% | 14 | 21.5% | 12 | 22.2% | 7 | 13.7% | 6 | 12.2% | 5 | 23.8% |
| – Down Syndrome | 17003 (7.3%) | 17 | 9.8% | 6 | 9.2% | 5 | 9.3% | 3 | 5.9% | 3 | 6.1% | 2 | 9.5% |
| – Patau syndrome/trisomy 13 | 1223 (0.5%) | 2 | 1.2% | 1 | 1.5% | 1 | 1.9% | 1 | 2.0% | 0 |  | 1 | 4.8% |
| – Edward syndrome/trisomy 18 | 3449 (1.5%) | 12 | 6.9% | 7 | 10.8% | 6 | 11.1% | 2 | 3.9% | 1 | 2.0% | 1 | 4.8% |
| – Turner syndrome | 1569 (0.7%) | 0 |  | 0 |  | 0 |  | 0 |  | 0 |  | 0 |  |
| – Klinefelter syndrome | 646 (0.3%) | 1 | 0.6% | 0 |  | 0 |  | 0 |  | 1 | 2.0% | 0 |  |

**Additional file number 4 continued: Description of congenital anomalies according to EUROCAT subgroup and by specific antiretroviral drug (n>15), after exposure to antiretroviral drugs, in 17 European registries between 1995 and 2019*.**

|  | **Unexposed to ARV** | **ARV** | | **Protease inhibitors** | | | | | |
| --- | --- | --- | --- | --- | --- | --- | --- | --- | --- |
|  |  |  | | **LPV** | | **NVP** | | **DRV** | |
| **Anomaly group** | 232418 | 173 | % | 48 | % | 17 | % | 15 | % |
| **Nervous system** | 20841 (9.0%) | 18 | 10.4% | 2 | 4.2% | 3 | 17.6% | 5 | 33.3% |
| – Neural Tube Defects | 7284 (3.1%) | 1 | 0.6% | 0 |  | 0 |  | 1 | 6.7% |
| – – Anencephalus and similar | 2078 (0.9%) | 0 |  | 0 |  | 0 |  | 0 |  |
| – – Encephalocele | 939 (0.4%) | 0 |  | 0 |  | 0 |  | 0 |  |
| – – Spina Bifida | 4267 (1.8%) | 1 | 0.6% | 0 |  | 0 |  | 1 | 6.7% |
| – Microcephaly | 2580 (1.1%) | 3 | 1.7% | 1 | 2.1% | 1 | 5.9% | 0 |  |
| – Hydrocephalus | 4463 (1.9%) | 3 | 1.7% | 1 | 2.1% | 0 |  | 1 | 6.7% |
| – Arhinencephaly/holoprosencephaly | 1000 (0.4%) | 0 |  | 0 |  | 0 |  | 0 |  |
| **Eye** | 4094 (1.8%) | 0 |  | 0 |  | 0 |  | 0 |  |
| – Anophthalmos/micropthalmos | 945 (0.4%) | 0 |  | 0 |  | 0 |  | 0 |  |
| – – Anophthalmos | 209 (0.1%) | 0 |  | 0 |  | 0 |  | 0 |  |
| – Congenital cataract | 1185 (0.5%) | 0 |  | 0 |  | 0 |  | 0 |  |
| – Congenital glaucoma | 304 (0.1%) | 0 |  | 0 |  | 0 |  | 0 |  |
| **Ear, face and neck** | 2703 (1.2%) | 2 | 1.2% | 1 | 2.1% | 0 |  | 0 |  |
| – Anotia | 335 (0.1%) | 0 |  | 0 |  | 0 |  | 0 |  |
| **Congenital heart defects** | 79676 (34.3%) | 73 | 42.2% | 25 | 52.1% | 7 | 41.2% | 4 | 26.7% |
| – Severe CHD § | 19866 (8.6%) | 26 | 15.0% | 6 | 12.5% | 2 | 11.8% | 2 | 13.3% |
| – Common arterial truncus | 649 (0.3%) | 2 | 1.2% | 0 |  | 1 | 5.9% | 1 | 6.7% |
| – Double outlet right ventricle § | 928 (0.4%) | 1 | 0.6% | 0 |  | 0 |  | 0 |  |
| – Transposition of great vessels | 2881 (1.2%) | 2 | 1.2% | 0 |  | 0 |  | 0 |  |
| – Single ventricle | 627 (0.3%) | 0 |  | 0 |  | 0 |  | 0 |  |
| – Ventricular septal defect (VSD) | 38634 (16.6%) | 29 | 16.8% | 11 | 22.9% | 2 | 11.8% | 2 | 13.3% |
| – Atrial septal defect (ASD) | 19076 (8.2%) | 12 | 6.9% | 4 | 8.3% | 1 | 5.9% | 0 |  |
| – Atrioventricular septal defect (AVSD) | 3773 (1.6%) | 6 | 3.5% | 2 | 4.2% | 0 |  | 0 |  |
| – Tetralogy of Fallot | 2841 (1.2%) | 7 | 4.0% | 2 | 4.2% | 0 |  | 1 | 6.7% |
| – Tricuspid atresia and stenosis | 547 (0.2%) | 1 | 0.6% | 0 |  | 0 |  | 0 |  |
| – Ebstein's anomaly | 408 (0.2%) | 0 |  | 0 |  | 0 |  | 0 |  |
| – Pulmonary valve stenosis | 3743 (1.6%) | 6 | 3.5% | 1 | 2.1% | 0 |  | 0 |  |
| – Pulmonary valve atresia | 840 (0.4%) | 2 | 1.2% | 1 | 2.1% | 1 | 5.9% | 0 |  |
| – Aortic valve atresia/stenosis § | 1532 (0.7%) | 1 | 0.6% | 1 | 2.1% | 0 |  | 0 |  |
| – Mitral valve anomalies | 1215 (0.5%) | 2 | 1.2% | 0 |  | 1 | 5.9% | 0 |  |
| – Hypoplastic left heart | 2158 (0.9%) | 3 | 1.7% | 0 |  | 0 |  | 0 |  |
| – Hypoplastic right heart § | 349 (0.2%) | 0 |  | 0 |  | 0 |  | 0 |  |
| – Coarctation of aorta | 3045 (1.3%) | 2 | 1.2% | 0 |  | 1 | 5.9% | 0 |  |
| – Aortic atresia/interrupted aortic arch | 293 (0.1%) | 0 |  | 0 |  | 0 |  | 0 |  |
| – Total anomalous pulm venous return | 443 (0.2%) | 0 |  | 0 |  | 0 |  | 0 |  |
| – PDA as only CHD in term infants (>=37 weeks) | 3329 (1.4%) | 2 | 1.2% | 0 |  | 0 |  | 0 |  |
| **Respiratory** | 2580 (1.1%) | 2 | 1.2% | 0 |  | 1 | 5.9% | 0 |  |
| – Choanal atresia | 701 (0.3%) | 0 |  | 0 |  | 0 |  | 0 |  |
| – Cystic adenomatous malf of lung § | 520 (0.2%) | 1 | 0.6% | 0 |  | 1 | 5.9% | 0 |  |
| **Oro-facial clefts** | 14186 (6.1%) | 1 | 0.6% | 0 |  | 0 |  |  |  |
| – Cleft lip with or without palate | 8221 (3.5%) | 0 |  | 0 |  | 0 |  | 0 |  |
| – Cleft palate | 5965 (2.6%) | 1 | 0.6% | 0 |  | 0 |  | 0 |  |
| **Digestive system** | 15653 (6.7%) | 17 | 9.8% | 5 | 10.4% | 0 |  | 1 | 6.7% |
| – Oesophageal atresia with or without tracheo-oesophageal fistula | 2166 (0.9%) | 5 | 2.9% | 2 | 4.2% | 0 |  | 0 |  |
| – Duodenal atresia or stenosis | 1041 (0.5%) | 0 |  | 0 |  | 0 |  | 0 |  |
| – Atresia or stenosis of other parts of small intestine | 616 (0.3%) | 2 | 1.2% | 2 | 4.2% | 0 |  | 0 |  |
| – Ano-rectal atresia and stenosis | 2714 (1.2%) | 1 | 0.6% | 0 |  | 0 |  | 0 |  |
| – Hirschsprung's disease | 997 (0.4%) | 0 |  | 0 |  | 0 |  | 0 |  |
| – Atresia of bile ducts | 290 (0.1%) | 1 | 0.6% | 0 |  | 0 |  | 0 |  |
| – Annular pancreas | 197 (0.1%) | 1 | 0.6% | 1 | 2.1% | 0 |  | 0 |  |
| – Diaphragmatic hernia | 2099 (0.9%) | 1 | 0.6% | 1 | 2.1% | 0 |  | 0 |  |
| **Abdominal wall defects** | 4140 (1.8%) | 4 | 2.3% | 1 | 2.1% | 0 |  | 0 |  |
| – Gastroschisis | 1668 (0.7%) | 2 | 1.2% | 1 | 2.1% | 0 |  | 0 |  |
| – Omphalocele | 2206 (1.0%) | 2 | 1.2% | 0 |  | 0 |  | 0 |  |
| **Urinary** | 31404 (13.5%) | 19 | 11.0% | 4 | 8.3% | 3 | 17.6% | 2 | 13.3% |
| – Bilateral renal agenesis including Potter syndrome | 848 (0.4%) | 0 |  | 0 |  | 0 |  | 0 |  |
| – Multicystic renal dysplasia | 2911 (1.3%) | 2 | 1.2% | 1 | 2.1% | 0 |  | 0 |  |
| – Congenital hydronephrosis | 10700 (4.6%) | 6 | 3.5% | 1 | 2.1% | 1 | 5.9% | 0 |  |
| – Bladder exstrophy and/or epispadia | 520 (0.2%) | 0 |  | 0 |  | 0 |  | 0 |  |
| – Posterior urethral valve and/or prune belly | 1289 (0.6%) | 1 | 0.6% | 1 | 2.1% | 0 |  | 0 |  |
| **Genital** | 19853 (8.5%) | 12 | 6.9% | 4 | 8.3% | 0 |  | 2 | 13.3% |
| – Hypospadias | 16523 (7.1%) | 8 | 4.6% | 4 | 8.3% | 0 |  | 0 |  |
| – Indeterminate sex | 483 (0.2%) | 0 |  | 0 |  | 0 |  | 0 |  |
| **Limb** | 39127 (16.8%) | 32 | 18.5% | 10 | 20.8% | 2 | 11.8% | 4 | 26.7% |
| – Limb reduction defects | 5319 (2.3%) | 4 | 2.3% | 1 | 2.1% | 1 | 5.9% | 0 |  |
| – Club foot - talipes equinovarus | 9723 (4.2%) | 4 | 2.3% | 1 | 2.1% | 0 |  | 1 | 6.7% |
| – Hip dislocation and/or dysplasia | 6619 (2.9%) | 4 | 2.3% | 1 | 2.1% | 0 |  | 0 |  |
| – Polydactyly | 10201 (4.4%) | 14 | 8.1% | 6 | 12.5% | 0 |  | 1 | 6.7% |
| – Syndactyly | 5279 (2.3%) | 4 | 2.3% | 1 | 2.1% | 0 |  | 0 |  |
| **Other anomalies/syndromes** |  |  |  |  |  |  |  |  |  |
| – Skeletal dysplasias § | 1320 (0.6%) | 0 |  | 0 |  | 0 |  | 0 |  |
| – Craniosynostosis | 2330 (1.0%) | 0 |  | 0 |  | 0 |  | 0 |  |
| – Congenital constriction bands/amniotic band | 422 (0.2%) | 1 | 0.6% | 0 |  | 0 |  | 0 |  |
| – Situs inversus | 615 (0.3%) | 1 | 0.6% | 1 | 2.1% | 0 |  | 0 |  |
| – Conjoined twins | 100 (0.04%) | 0 |  | 0 |  | 0 |  | 0 |  |
| – Congenital skin disorders | 1912 (0.8%) | 0 |  | 0 |  | 0 |  | 0 |  |
| – VATER/VACTERL | 309 (0.1%) | 0 |  | 0 |  | 0 |  | 0 |  |
| – Vascular disruption anomalies § | 5945 (2.6%) | 6 | 3.5% | 5 | 10.4% | 1 | 5.9% | 0 |  |
| – Lateral anomalies § | 1510 (0.7%) | 3 | 1.7% | 1 | 2.1% | 0 |  | 0 |  |
| – Teratogenic syndromes with malformations § | 990 (0.4%) | 4 | 2.3% | 0 |  | 1 | 5.9% | 1 | 6.7% |
| – Fetal alcohol syndrome | 365 (0.2%) | 2 | 1.2% | 0 |  | 1 | 5.9% | 0 |  |
| – Valproate syndrome § | 58 (0.02%) | 0 |  | 0 |  | 0 |  | 0 |  |
| – Maternal infections resulting in malformations | 534 (0.2%) | 2 | 1.2% | 0 |  | 1 | 5.9% | 1 | 6.7% |
| – Genetic syndromes + microdeletions | 4616 (2.0%) | 5 | 2.9% | 0 |  | 1 | 5.9% | 0 |  |
| **Chromosomal** | 28454 (12.2%) | 31 | 17.9% | 10 | 20.8% | 3 | 17.6% | 4 | 26.7% |
| – Down Syndrome | 17003 (7.3%) | 17 | 9.8% | 6 | 12.5% | 1 | 5.9% | 1 | 6.7% |
| – Patau syndrome/trisomy 13 | 1223 (0.5%) | 2 | 1.2% | 1 | 2.1% | 0 |  | 0 |  |
| – Edward syndrome/trisomy 18 | 3449 (1.5%) | 12 | 6.9% | 2 | 4.2% | 2 | 11.8% | 3 | 20.0% |
| – Turner syndrome | 1569 (0.7%) | 0 |  | 0 |  | 0 |  | 0 |  |
| – Klinefelter syndrome | 646 (0.3%) | 1 | 0.6% | 0 |  | 0 |  | 0 |  |

*ABC: abacavir; ATV: atazanavir; FTC: emtricitabine; LPV: lopinavir; NVP: nevirapine; TDF: tenofovir; ZDV: zidovudine; 3TC: lamivudine.*

**All registries did not cover the same study period due to difference in data availability at the time of study analysis as of October 2023.*

**Additional file number 5: Description of significant signals observed after confirmed exposure during the first trimester of pregnancy to zidovudine (signal verification analysis), tenofovir, emtricitabine, lopinavir and darunavir (signal detection analysis).**

**Additional file number 5A: Description of congenital heart defects cases observed after confirmed exposure during the first trimester of pregnancy to zidovudine (signal verification analysis) and lamivudine (signal detection analysis).**

| **Cases** | **Zidovudine** | **Lamivudine** | **Other antiretroviral drugs** | **Congenital malformations of the circulatory system** | **Other congenital anomalies** |
| --- | --- | --- | --- | --- | --- |
| **ID1** | Zidovudine | Lamivudine |  | Ventricular septal defect |  |
| **ID2** | Zidovudine |  |  | Atrioventricular septal defect and other specified congenital anomalies of heart |  |
| **ID3** | Zidovudine | Lamivudine | Nevirapine | Congenital pulmonary valve stenosis |  |
| **ID4** | Zidovudine | Lamivudine | Nevirapine, indinavir | Ventricular septal defect |  |
| **ID5** | Zidovudine |  |  | Hypoplastic left heart syndrome |  |
| **ID5** | Zidovudine | Lamivudine |  | Stenosis of pulmonary artery |  |
| **ID6** | Zidovudine |  | Abacavir | Ventricular septal defect |  |
| **ID7** | Zidovudine |  |  | Atrial septal defect |  |
| **ID8** | Zidovudine | Lamivudine |  | Ventricular septal defect, stenosis of pulmonary artery, atrial septal defect, and hypertrophic right anterior wall |  |
| **ID9** | Zidovudine | Lamivudine | Abacavir | Stenosis of pulmonary artery and atrial septal defect |  |
| **ID10** | Zidovudine | Lamivudine | Fosamprenavir/ritonavir | Tetralogy of Fallot |  |
| **ID11** | Zidovudine |  |  | Stenosis of pulmonary artery and atrial septal defect |  |
| **ID12** | Zidovudine | Lamivudine |  | Ventricular septal defect and atrial septal defect |  |
| **ID13** | Zidovudine | Lamivudine |  | Atrial septal defect |  |
| **ID14** | Zidovudine |  |  | Atrial septal defect |  |
| **ID15** | Zidovudine | Lamivudine |  | Atrial septal defect |  |
| **ID16** | Zidovudine | Lamivudine |  | Atrial septal defect | Pyelo-calicielle dilatation, bifid kidney, ureter duplication and congenital vesico-ureterorenal reflux |
| **ID17** |  | Lamivudine | Abacavir, efavirenz | Ventricular septal defect and atrial septal defect |  |
| **ID18** |  | Lamivudine | Abacavir | Ventricular septal defect |  |
| **ID19** |  | Lamivudine | Abacavir | Hypoplasia of aorta | Congenital absence, atresia and stenosis of ileum and congenital renal calculi |
| **ID20** |  | Lamivudine | Abacavir, darunavir/  ritonavir | Ventricular septal defect |  |
| **ID21** |  |  | Combination not specified | Ventricular septal defect |  |
| **ID22** |  |  | Combination not specified | Congenital pulmonary valve stenosis |  |

**Additional file number 5B: Description of congenital anomaly of the nervous system cases observed after confirmed exposure during the first trimester of pregnancy to darunavir (signal detection analysis).**

| **Cases** | **Darunavir** | **Other antiretroviral drugs** | **Congenital anomaly of the nervous system** | **Other congenital anomalies** |
| --- | --- | --- | --- | --- |
| **ID1** | Darunavir | Tenofovir, emtricitabine | Pachygyria | Micromyelia of the four members, bilateral cortical renal cysts, nephromegaly and hypogenitalism |
| **ID2** | Darunavir | Tenofovir, emtricitabine | Malformations of aqueduct of Sylvius and hypoplasia of cerebellum |  |
| **ID3** | Darunavir | Tenofovir, emtricitabine, raltegravir | Hypoplasia of the corpus callosum | Volvulus of the small intestine |
| **ID4** | Darunavir | Tenofovir, emtricitabine | Lumbo-sacral spina bifida with hydrocephalus |  |

**Additional file number 5C: Description of polydactyly cases observed after confirmed exposure during the first trimester of pregnancy to tenofovir, emtricitabine and lopinavir (signal detection analysis).**

| **Cases** | **Tenofovir** | **Emtricitabine** | **Lopinavir** | **Other antiretroviral drugs** | **Polydactyly or other congenital anomaly of the musculoskeletal system** | **Other congenital anomalies** |
| --- | --- | --- | --- | --- | --- | --- |
| **ID1** | Tenofovir |  | Lopinavir/ ritonavir | Zidovudine, abacavir | Polydactyly: extra left finger |  |
| **ID2** | Tenofovir | Emtricitabine | Lopinavir/ ritonavir |  | Polydactyly: extra fingers | Congenital absence, atresia and stenosis of duodenum, annular pancreas and situs inversus |
| **ID3** | Tenofovir | Emtricitabine |  | Nevirapine | Polydactyly: extra bilateral fingers |  |
| **ID4** | Tenofovir | Emtricitabine |  | Darunavir/ritonavir | Polydactyly: extra right finger |  |
| **ID5** | Tenofovir | Emtricitabine |  | Nevirapine | Polydactyly: extra bilateral fingers |  |
| **ID6** | Tenofovir | Emtricitabine |  | Darunavir/ritonavir, raltegravir | Polydactyly: extra bilateral fingers |  |
| **ID7** |  |  | Lopinavir/ ritonavir | Zidovudine, lamivudine | Polydactyly: extra right finger and extra bilateral toes, Webbed fingers |  |
| **ID8** |  |  | Lopinavir/ ritonavir | Abacavir, lamivudine | Polydactyly: extra post-axial fingers | Tetralogy of Fallot |

**Additional file number 6**

**Reporting odds ratio estimated in the sensitivity analysis including 171 cases, for confirmation of previous signals between the occurrence of congenital anomalies and exposure to antiretroviral drugs.**

|  |  |  |  |  |  |
| --- | --- | --- | --- | --- | --- |
| Signal verification analysis | Cases (n, %) | Non-cases (n, %) | aROR [95%CI]* | Non-cases (n, %) | aROR [95%CI]* |
|  | Congenital heart defects (n=79,549) | Other non-genetic anomalies  (n=120,640) |  | Genetic control group (n=32,985) |  |
| ZDV | 23 (0.03%) | 18 (0.01%) | **2.51 [1.35-4.66]** | 7 (0.02%) | 1.97 [0.82-4.73] |
| *Adjustment on registries and maternal age | |  |  |  |  |

| **Reporting odds ratio estimated in the sensitivity analysis including 171 cases, for detection of new signals between the occurrence of congenital anomalies and exposure to antiretroviral drugs.** | | | | | | | |
| --- | --- | --- | --- | --- | --- | --- | --- |
| Signal detection analyses | Cases (n, %) | Non-cases (n, %) |  |  | Non-cases (n, %) |  |  |
|  | Nervous system  (n=20,753) | Other non-genetic anomalies  (n=179,436) | ROR [95%CI] | p-value with Bonferroni correction (p<0.0015) | Genetic control group (n=32,985) | ROR [95%CI] | p-value with Bonferroni correction (p<0.0015) |
| TDF | 6 (0.03%) | 45 (0.02%) | 1.33 [0.57-3.12] | 0.5093 | 13 (0.04%) | 0.84 [0.32-2.21] | 0.7226 |
| FTC | 5 (0.03%) | 37 (0.02%) | 1.35 [0.53-3.44] | 0.5285 | 12 (0.04%) | 0.75 [0.27-2.14] | 0.5958 |
| DRV | 4 (0.02%) | 10 (0.01%) | 3.98 [1.25-12.25] | 0.0192 | 4 (0.01%) | 1.81 [0.45-7.24] | 0.4017 |
|  | Congenital heart defects (CHD)  (n=79,549) | Other non-genetic anomalies  (n=120,640) | ROR [95%CI] | p-value with Bonferroni correction (p<0.0015) | Genetic control group (n=32,985) | ROR [95%CI] | p-value with Bonferroni correction (p<0.0015) |
| TDF | 19 (0.03%) | 32 (0.02%) | 1.10 [0.62-1.93] | 0.7551 | 13 (0.04%) | 0.68 [0.34-1.39] | 0.2913 |
| FTC | 16 (0.02%) | 26 (0.02%) | 1.14 [0.61-2.12] | 0.6889 | 12 (0.04%) | 0.62 [0.30-1.32] | 0.2168 |
| 3TC | 21 (0.03%) | 20 (0.02%) | 1.93 [1.05-3.57] | 0.0349 | 9 (0.03%) | 1.09 [0.50-2.38] | 0.8280 |
| LPV | 17 (0.01%) | 18 (0.01%) | 1.95 [1.01-3.78] | 0.0484 | 10 (0.03%) | 0.84 [0.39-1.82] | 0.8628 |
| ABC | 8 (0.01%) | 7 (0.01%) | 2.10 [0.76-5.80] | 0.1506 | 5 (0.02%) | 0.74 [0.24-2.25] | 0.5944 |
| ATV | 6 (0.01%) | 7 (0.01%) | 1.58 [0.53-4.70] | 0.4121 | 4 (0.01%) | 0.70 [0.20-2.49] | 0.5835 |
| DRV | 5 (0.01%) | 9 (0.01%) | 1.02 [0.34-3.05] | 0. 9674 | 4 (0.01%) | 1.71 [0.46-6.37] | 0.4241 |
|  | Severe CHD  (n=19,840) | Other non-genetic anomalies excluding others CHD  (n=120,640) | ROR [95%CI] | p-value with Bonferroni correction (p<0.0015) | Genetic control group (n=32,985) | ROR [95%CI] | p-value with Bonferroni correction (p<0.0015) |
| TDF | 6 (0.04%) | 45 (0.02%) | 1.53 [0.65-3.58] | 0.3287 | 13 (0.04%) | 0.95 [0.36-2.49] | 0.9100 |
| FTC | 6 (0.04%) | 36 (0.02%) | 1.91 [0.80-4.52] | 0.1437 | 12 (0.04%) | 1.02 [0.38-2.73] | 0.9620 |
|  | Ventricular septal defect (n=38,575) | Other non-genetic anomalies excluding others CHD  (n=120,640) | ROR [95%CI] | p-value with Bonferroni correction (p<0.0015) | Genetic control group (n=32,985) | ROR [95%CI] | p-value with Bonferroni correction (p<0.0015) |
| TDF | 5 (0.01%) | 46 (0.03%) | 0.51 [0.20-1.28] | 0.1500 | 13 (0.04%) | 0.36 [0.13-1.01] | 0.0511 |
| FTC | 5 (0.01%) | 37 (0.02%) | 0.63 [0.23-1.60] | 0.3300 | 12 (0.04%) | 0.39 [0.14-1.10] | 0.0753 |
| 3TC | 9 (0.02%) | 32 (0.02%) | 1.31 [0.62-2.74] | 0.4783 | 9 (0.03%) | 0.93 [0.37-2.34] | 0.8773 |
| ZDV | 8 (0.02%) | 33 (0.02%) | 1.13 [0.52-2.45] | 0.7513 | 7 (0.02%) | 1.06 [0.39-2.93] | 0.9066 |
| LPV | 8 (0.02%) | 27 (0.02%) | 1.38 [0.63-3.03] | 0.4269 | 10 (0.03%) | 0.75 [0.29-1.89] | 0.5350 |
| ABC | 4 (0.01%) | 11 (0.01%) | 1.69 [0.54-5.31] | 0.3674 | 5 (0.02%) | 0.75 [0.20-2.78] | 0.6609 |
|  | Digestive system  (n=15,637) | Other non-genetic anomalies  (n=184,552) | ROR [95%CI] | p-value with Bonferroni correction (p<0.0015) | Genetic control group (n=32,985) | ROR [95%CI] | p-value with Bonferroni correction (p<0.0015) |
| TDF | 4 (0.03%) | 47 (0.03%) | 1.15 [0.42-3.19] | 0.7849 | 13 (0.04%) | 0.73 [0.24-2.24] | 0.5845 |
| FTC | 5 (0.04%) | 37 (0.02%) | 1.82 [0.71-4.62] | 0.2109 | 12 (0.04%) | 0.99 [0.35-2.81] | 0.9856 |
| 3TC | 4 (0.03%) | 37 (0.02%) | 1.46 [0.52-4.08] | 0.4763 | 9 (0.03%) | 1.06 [0.33-3.43] | 0.9264 |
|  | Genital system  (n=19,797) | Other non-genetic anomalies  (n=180,392) | ROR [95%CI] | p-value with Bonferroni correction (p<0.0015) | Genetic control group (n=32,985) | ROR [95%CI] | p-value with Bonferroni correction (p<0.0015) |
| TDF | 7 (0.04%) | 44 (0.02%) | 1.51 [0.68-3.35] | 0.3107 | 13 (0.04%) | 0.93 [0.37-2.33] | 0.8765 |
| FTC | 6 (0.03%) | 36 (0.02%) | 1.58 [0.67-3.76] | 0.2967 | 12 (0.04%) | 1.68 [0.59-4.77] | 0.7723 |
|  | Urinary system  (n=31,381) | Other non-genetic anomalies  (n=168,805) | ROR [95%CI] | p-value with Bonferroni correction (p<0.0015) | Genetic control group (n=32,985) | ROR [95%CI] | p-value with Bonferroni correction (p<0.0015) |
| TDF | 9 (0.03%) | 42 (0.02%) | 1.23 [0.60-2.52] | 0.5759 | 13 (0.04%) | 0.77 [0.33-1.80] | 0.5467 |
| FTC | 7 (0.02%) | 37 (0.02%) | 1.15 [0.51-2.59] | 0.7292 | 12 (0.04%) | 0.65 [0.26-1.64] | 0.3595 |
|  | Congenital hydronephrosis (n=10,697) | Other non-genetic anomalies excluding others urinary congenital anomalies (n=168,805) | ROR [95%CI] | p-value with Bonferroni correction (p<0.0015) | Genetic control group (n=32,985) | ROR [95%CI] | p-value with Bonferroni correction (p<0.0015) |
| TDF | 4 (0.04%) | 47 (0.02%) | 1.57 [0.57-4.34] | 0.3878 | 13 (0.04%) | 0.98 [0.32-3.01] | 0.9720 |
|  | Limb defects  (n=39,186) | Other non-genetic anomalies  (n=161,003) | ROR [95%CI] | p-value with Bonferroni correction (p<0.0015) | Genetic control group (n=32,985) | ROR [95%CI] | p-value with Bonferroni correction (p<0.0015) |
| TDF | 12 (0.03%) | 39 (0.02%) | 1.39 [0.73-2.65] | 0.3234 | 13 (0.04%) | 0.84 [0.38-1.83] | 0.6549 |
| FTC | 11 (0.03%) | 31 (0.02%) | 1.60 [0.80-3.18] | 0.1810 | 12 (0.04%) | 0.83 [0.37-1.88] | 0.6560 |
| 3TC | 5 (0.01%) | 36 (0.02%) | 1.46 [0.52-4.08] | 0.3265 | 9 (0.03%) | 0.50 [0.17-1.50] | 0.2185 |
| LPV | 8 (0.02%) | 27 (0.02%) | 1.33 [0.61-2.94] | 0.3805 | 10 (0.03%) | 0.73 [0.29-1.84] | 0.4979 |
| ABC | 3 (0.01%) | 12 (0.01%) | 1.13 [0.32-4.00] | 0.8493 | 5 (0.02%) | 0.55 [0.13-2.29] | 0.4108 |
| NVP | 6 (0.02%) | 10 (0.01%) | 2.72 [0.99-7.48] | 0.0525 | 4 (0.01%) | 1.35 [0.38-4.79] | 0.6395 |
| DRV | 5 (0.01%) | 9 (0.01%) | 2.52 [0.85-7.51] | 0.0975 | 4 (0.01%) | 1.13 [0.30-4.22] | 0.8534 |
|  | Polydactyly  (n=10,192) | Other non-genetic anomalies excluding others limb defects  (n=161,003) | ROR [95%CI] | p-value with Bonferroni correction (p<0.0015) | Genetic control group (n=32,985) | ROR [95%CI] | p-value with Bonferroni correction (p<0.0015) |
| TDF | 7 (0.07%) | 44 (0.02%) | 3.18 [1.43-7.06] | 0.0045 | 13 (0.04%) | 1.86 [0.74-4.67] | 0.1848 |
| FTC | 5 (0.05%) | 37 (0.02%) | 2.71 [1.07-6.88] | 0.0363 | 12 (0.04%) | 1.44 [0.51-4.09] | 0.4938 |
| LPV | 4 (0.04%) | 31 (0.02%) | 2.58 [0.91-7.31] | 0.0738 | 10 (0.03%) | 1.38 [0.43-4.41] | 0.5847 |

*ABC: abacavir; ATV: atazanavir; CHD: congenital heart defects; FTC: emtricitabine; LPV: lopinavir; NVP: nevirapine; TDF: tenofovir; ZDV: zidovudine; 3TC: lamivudine; 95%CI: 95% confidence intervals.*
